# Supplementary material for: A qualitative study of graduate student emotional and cognitive processing of unexpected (chance) events
Source: PLoS One. 2025 Aug 28;20(8):e0331182. doi: 10.1371/journal.pone.0331182 (PMC12393738; doi:10.1371/journal.pone.0331182)
Supplement: S1 Appendix — (PDF) [file pone.0331182.s001.pdf]

Supplemental Table 1: *Interview questions*

**1**	In your survey, you mentioned that your original career intention(s) was/were "x" Can you tell me why you were interested in that career?	
	Follow-up questions	What did you think you needed to accomplish to get the job?<br>How capable did you feel to do that type of job or to get that job?
**2**	In your survey response you stated that "a,b,c, etc.." chance events occurred and had a high or slightly high impact on your career intentions. Can you place each of these events in order from those that had the lowest to the highest impact on your career goals?	
	Follow-up questions	Can you think of a specific chance event that occurred in "x" category?<br>Where would you place this specific event on the Likert scale?
**3**	Can you tell me more about the specific event that you felt was most impactful to your career intentions?	
	Follow-up questions	At what point did this happen in graduate school?<br>Can you describe the event to me?<br>What was happening in your graduate life before this occurred?<br>How did you feel about the event as it happened?<br>How did you feel about the event a few months after it happened? Currently?<br>Did you feel you had any control over the event?<br>Why do you think it had an impact on your career intention?
