# Supplementary material for: A qualitative study of graduate student emotional and cognitive processing of unexpected (chance) events
Source: PLoS One. 2025 Aug 28;20(8):e0331182. doi: 10.1371/journal.pone.0331182 (PMC12393738; doi:10.1371/journal.pone.0331182)
Supplement: S2 Appendix — (PDF) [file pone.0331182.s002.pdf]

Khaki's Appraisal Theory Story Arc

CONTEXT

"I had just passed that exam in February and was actually right after…if you're a PhD student knows that's incredibly stressful…shortly after …COVID was going on…So basically, in the two months, I was working from home… reading papers, adjusting things like trying to come up with new…experimental designs and ideas and then have that committee meeting…"

CHANCE EVENT

"…It was literally the day after my committee meeting. (laughing)… literally the day after my committee meeting, was when I found out my, my PI, I took another job…It was a complete surprise…"

PRIMARY APPRAISAL

"…I was [pause] incredibly upset. Just because you come into graduate school thinking okay, I'm going to spend five years here... I had a really good community, both in my personal life and my professional life. And then I realized all of that was going to change."

SECONDARY APPRAISAL

"…it only took me about five hours to decide I was moving. And the ultimate reason for that was, I knew this would be better for my career in the long run if I did go… I definitely knew it was an opportunity that I was given for a reason…"

REAPPRAISAL

"...I would say it's kind of a roller coaster. Where some days I was really happy and some days I was… I was sad or stressed and I think because a situation like moving is so, [PAUSE] Is tied to your personal life as well. I think that's what pushed a lot of the roller coaster [PAUSE]…"

OUTCOME

"Prior to moving, I felt like that was not ever something I could do. But now I feel like I have the confidence to be like, Okay, I can, you know, teach myself, I can go into that situation. And I can teach myself how to do that. Because I have taught myself not only my own research thus far…I've just gained a lot of confidence…I can do anything that I need to learn how to do like, it's possible."

Story Arc Component	Appraisal Theory Component	Khaki’s Written Story Component
**Exposition** *(characters/setting)*	**Participant Context before event**	Khaki had just passed her passed Preliminary exams for her PhD program. She mentioned initial stress from these initial exams and that she was asked to have another committee meeting to clarify aspects of her work.
**Rising Action** (*Series of events)*	**Chance Event**	At the end of her committee meeting, Khaki’s advisor told her she had taken a new job at another university and would be leaving in the next year. Khaki was given the option to stay where she was or move universities with her advisor.
**Climax** *(Turning point)*	**Primary & Secondary Appraisal**	Khaki was initially upset when told, highlighting an initial negative primary appraisal. She reflected on the community she built and never expected to have to make this type of decision. Khaki reflected on this but quickly thought about the potential for building a bigger network for her career, considering opportunities to collaborate with others, thus highlighting a secondary appraisal where she evaluated options and weighed the pros and cons to moving. She ultimately decides to move with her advisor.
**Falling Action** *(movement to a new normal)*	**Reappraisal**	Khaki spoke of multiple reevaluations where there are times she is overwhelmed and stressed with moving and setting up a lab but ends with seeing this unexpected move providing opportunities to build skillsets.
**Resolution**	**Outcome**	Khaki finds a new sense of belonging and states her confidence grew. She now sees herself in new positions and is confident to do them successfully.
