# Supplementary material for: A qualitative study of graduate student emotional and cognitive processing of unexpected (chance) events
Source: PLoS One. 2025 Aug 28;20(8):e0331182. doi: 10.1371/journal.pone.0331182 (PMC12393738; doi:10.1371/journal.pone.0331182)
Supplement: S3 Appendix — (PDF) [file pone.0331182.s003.pdf]

Composite Narrative for Graduate Students Who Changed Their Career Intentions

All of the graduate students who changed their career intentions discussed a consistent and underlying level of stress and anxiety in their programs before the chance event. They often struggled with self-doubt about their abilities or skills, questioned the importance of their work, or felt increased academic pressure. This group also highlighted personal stressors that they were managing outside of school. **This highlights the initial stressors theme.** For example, Violet said, *"Starting graduate school, in a pandemic, is a certain level of stress, right…stuff with my brother was happening at the same time…Also going through a really traumatic breakup."*

While participants in this group had various stressors, they appeared to expect these types of experiences in graduate school and knew how to manage them. However, chance events represented something unexpected, and participants initially viewed those events negatively because of this. The chance events for these participants included unexpected negative feedback about research progress, health diagnoses, unexpected job openings, and the COVID-19 pandemic. These events were initially viewed as significant barriers to their career goals. **Their views support the emotional toll theme that was found at the primary appraisal stage of the Appraisal Theory Story Arc.** For example, Beige said in response to her chance event (an unexpected job opening), *"I felt like I am missing out. I feel I'm trapped in this thing that I am doing...My committee is gonna be mad at me or think I'm like, trying to flake out or I don't know…I wish I chose a different path in my life, so that I could apply for this job."*

Often, in response to these chance events, participants reached out for support and advice from peers, friends, family, and occasionally mentors or faculty members. **This provided evidence of the help seeking theme at the secondary appraisal stage.** Violet stated, "*I began kind of reaching out for help. Talking to my cohort…So reaching out to my peers was really helpful in that. I had a long discussion with my advisor…And he was like, it was really a lot, all my friends and family that were like, give yourself grace. And I was like, Okay, I guess I deserve that."* This network of support was seen by the participants as helping them navigate the uncertainty of the events.

As time passed after the event, participants in this group talked about beginning to see them with a different perspective. Many participants recognized struggles with their mental health that were previously unacknowledged. **These highlight the theme of realizations at the reappraisal stage of the narrative.** Coffee shared, *"…it made me realize that it was important for me to maybe take my mental health seriously... I didn't really do that my first three years..."* Participants began to reanalyze their personal values and well-being in relation to their careers.

Participants in this group talked about this time after reconciling the chance event as giving them opportunities to explore new opportunities and new paths that better

suited their needs and aspirations. The chance events, initially perceived as setbacks, were talked about as catalysts for growth. Participants expressed advocating for themselves and pursuing careers that offered greater fulfillment and alignment with their values. **This supported the growth theme at the outcome stage (resolution) of this group's narrative**. For example, Beige says, *"I do feel now that I'm more able to speak up about things that are making me uncomfortable, or situations where I'm unhappy and be able to act on things more, rather than just wallowing in my emotions…"*

Composite Narrative for Graduate Students Who Did Not Change Their Career Intentions

Like those who did change their career intentions, graduate students in this group mentioned that they struggled with self-doubt, increased academic pressure, or questioned the importance of their research while in their programs before the chance event occurred. **This highlights the initial stressor theme which was prevalent at the context stage (i.e., exposition) of participants' narratives.** Yellow, for example, said, *"…there are days where it feels very, within my reach, and then others where it's like, this is a pipe dream of the wildest imaginations…I can't even get salt to go into water. And it's like, Ooh, maybe research is too hard…"*

When unexpected events like the COVID-19 pandemic, unexpected negative feedback, or shifts in research projects occurred, participants talked about these things generating negative feelings and sowing doubts about their careers and identities, **marking the emotional toll theme occurring at the primary appraisal stage of narratives**. For example, Pearl said in response to unexpected negative feedback, *"…it just really hurt because I care a lot about, not necessarily what students think of me, but that I am being fair, and being supportive, and just their own emotional state in a class or if the class is enjoyable for them…I questioned my ability to be a good educator and TA for a long time after that, like, I had bought a sweater I was really excited about that talked about it said like, teach coffee sleep or something. It was supposed to be like, This is my life…I didn't wear it for a long time because…I don't feel like I deserve to wear this because I really felt like I was really hurting my students."*

Faced with uncertainty about their next steps, participants often talked about self-reflection, assessing their skills, knowledge, or feelings to help them decide how to move forward. They used different strategies like self-talk, suppressing emotions, or assessing pros and cons of perceived solutions. **This demonstrated the self-reliance theme at the secondary appraisal stage of the narrative.** For example, in response to a chance event that caused her to feel uncertain and anxious about her future, Khaki assessed her potential solutions, saying *"I had to decide that going somewhere new that had more people doing what I'm interested in and more connections I could make,*

and more collaboration opportunities was what was best for me and my future as scientist..."

Over time, participants talked about continuing to use these strategies (i.e., self-talk, emotion suppression, assessment of pros and cons, etc.) and expressed how the chance events had provided opportunities to build resilience and enhance their skills in research or teaching. Specifically, all five participants in this group mentioned building skills or confidence to do hard things. **This demonstrated the resilience theme at the reappraisal stage.** For example, Bronze said, *"I think it showed me that I can do super monotonous work for very long periods of time. And like…I hate wet lab work. But I was like, I guess I can do this… it showed me that I had grit that I could get things done."*

Ultimately, all participants in this group talked about coming to terms with the chance event and the negative feelings it generated. They discussed ways they had come to see it as a positive factor influencing skill development and helping them reach their career goals more confidently. **This highlights the growth theme at the outcome (i.e., resolution) of participant narratives.** For example, Pearl says, *"It's a learning experience is how I'm viewing it…And so I think now I'm, I'm a little careful with how I comment, but now I also let my students know that if you're getting this vibe from my comments, that's not intentional…"*
