# Supplementary material for: A qualitative study of graduate student emotional and cognitive processing of unexpected (chance) events
Source: PLoS One. 2025 Aug 28;20(8):e0331182. doi: 10.1371/journal.pone.0331182 (PMC12393738; doi:10.1371/journal.pone.0331182)
Supplement: S4 Appendix — (PPTX) [file pone.0331182.s004.pptx]

## Slide 1
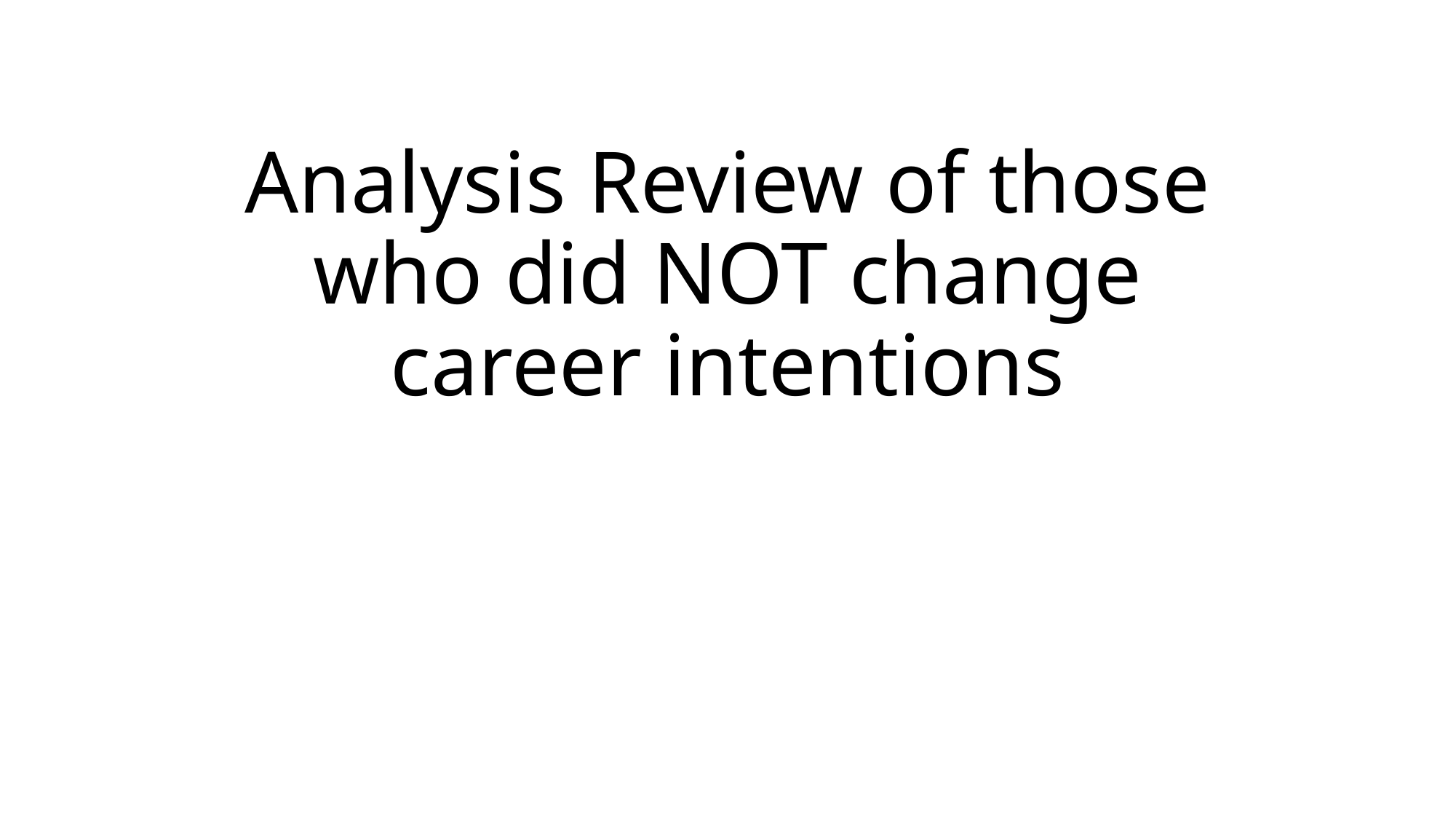

# Analysis Review of those who did NOT change career intentions

## Slide 2
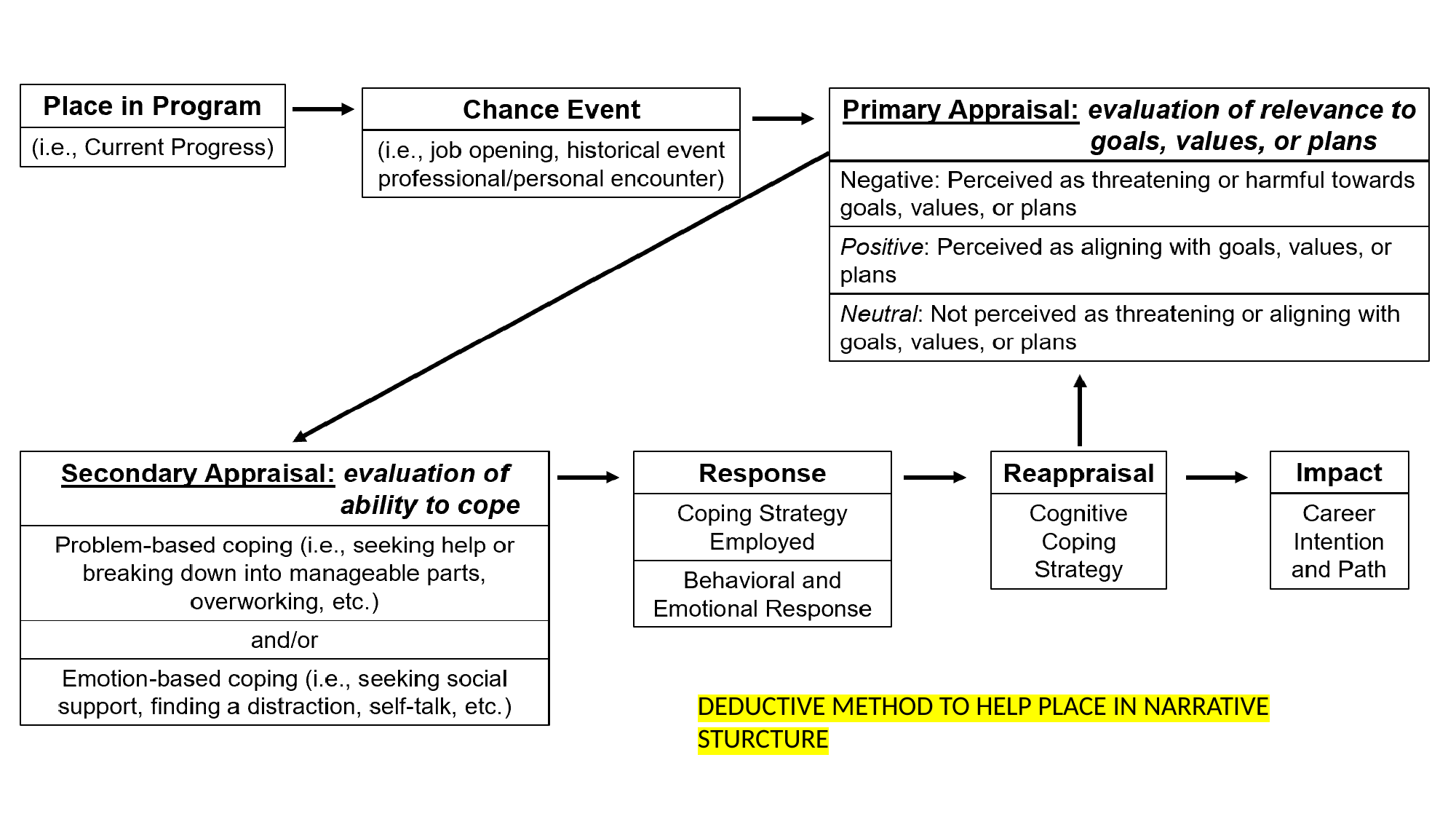

DEDUCTIVE METHOD TO HELP PLACE IN NARRATIVE STURCTURE

## Slide 3
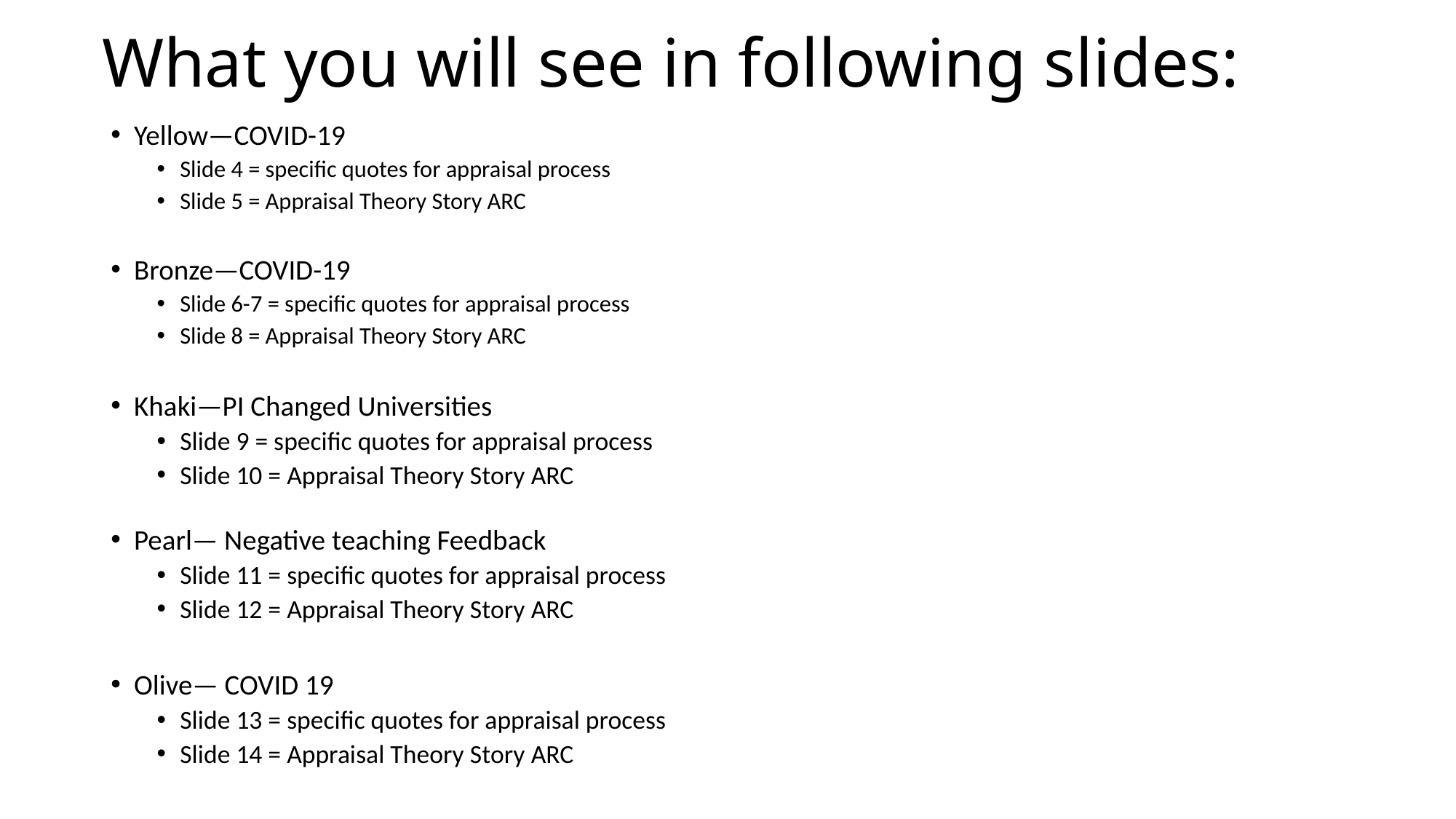

# What you will see in following slides:
Yellow—COVID-19
Slide 4 = specific quotes for appraisal process
Slide 5 = Appraisal Theory Story ARC
Bronze—COVID-19
Slide 6-7 = specific quotes for appraisal process
Slide 8 = Appraisal Theory Story ARC
Khaki—PI Changed Universities
Slide 9 = specific quotes for appraisal process
Slide 10 = Appraisal Theory Story ARC
Pearl— Negative teaching Feedback
Slide 11 = specific quotes for appraisal process
Slide 12 = Appraisal Theory Story ARC
Olive— COVID 19
Slide 13 = specific quotes for appraisal process
Slide 14 = Appraisal Theory Story ARC

## Slide 4
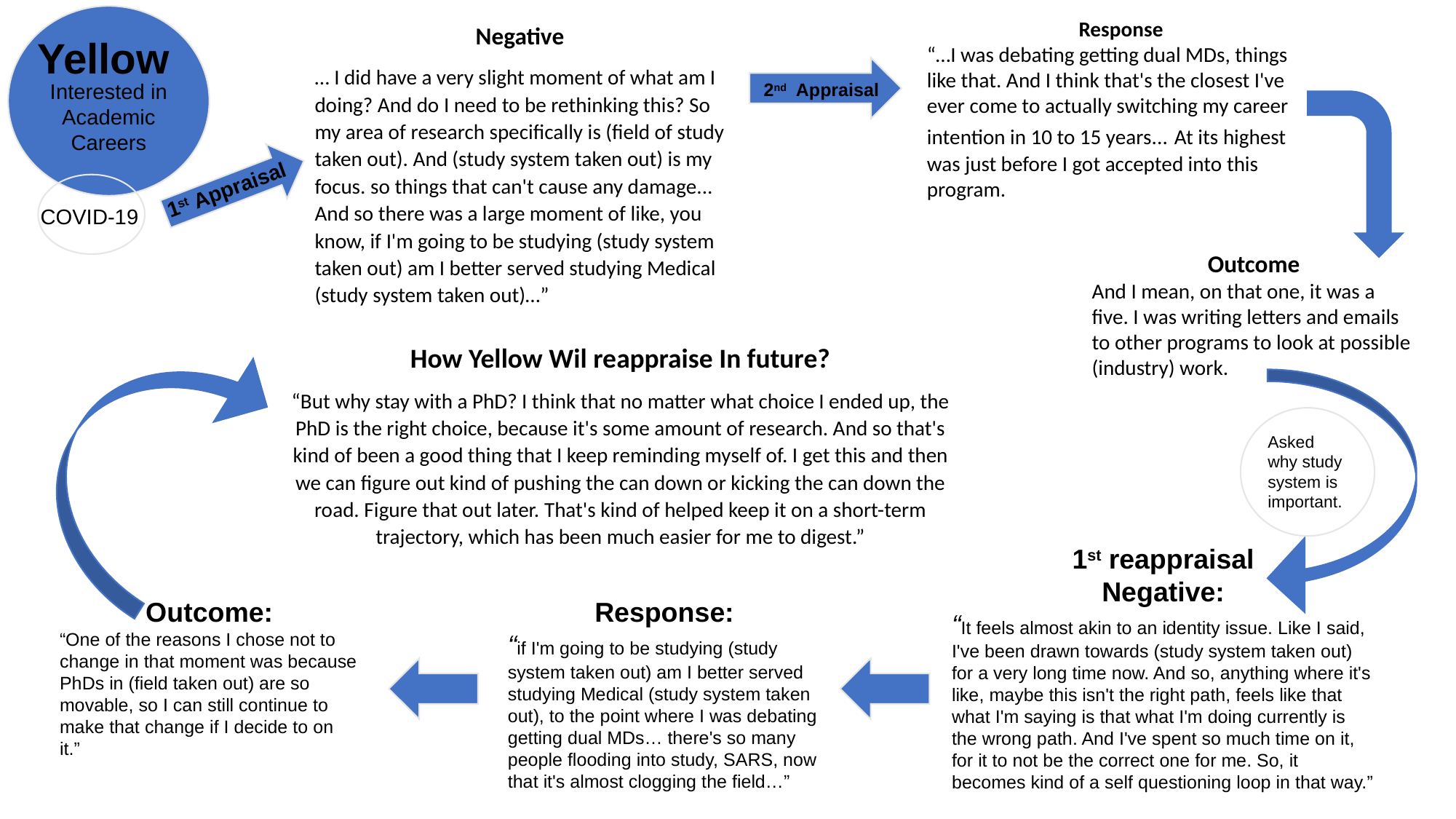

Response
“…I was debating getting dual MDs, things like that. And I think that's the closest I've ever come to actually switching my career intention in 10 to 15 years... At its highest was just before I got accepted into this program.
Negative
… I did have a very slight moment of what am I doing? And do I need to be rethinking this? So my area of research specifically is (field of study taken out). And (study system taken out) is my focus. so things that can't cause any damage... And so there was a large moment of like, you know, if I'm going to be studying (study system taken out) am I better served studying Medical (study system taken out)…”
Yellow
Interested in Academic Careers
2nd Appraisal
1st Appraisal
COVID-19
Outcome
And I mean, on that one, it was a five. I was writing letters and emails to other programs to look at possible (industry) work.
How Yellow Wil reappraise In future?
“But why stay with a PhD? I think that no matter what choice I ended up, the PhD is the right choice, because it's some amount of research. And so that's kind of been a good thing that I keep reminding myself of. I get this and then we can figure out kind of pushing the can down or kicking the can down the road. Figure that out later. That's kind of helped keep it on a short-term trajectory, which has been much easier for me to digest.”
Asked why study system is important.
1st reappraisal
Negative:
“It feels almost akin to an identity issue. Like I said, I've been drawn towards (study system taken out) for a very long time now. And so, anything where it's like, maybe this isn't the right path, feels like that what I'm saying is that what I'm doing currently is the wrong path. And I've spent so much time on it, for it to not be the correct one for me. So, it becomes kind of a self questioning loop in that way.”
Outcome:
“One of the reasons I chose not to change in that moment was because PhDs in (field taken out) are so movable, so I can still continue to make that change if I decide to on it.”
Response:
“if I'm going to be studying (study system taken out) am I better served studying Medical (study system taken out), to the point where I was debating getting dual MDs… there's so many people flooding into study, SARS, now that it's almost clogging the field…”

## Slide 5
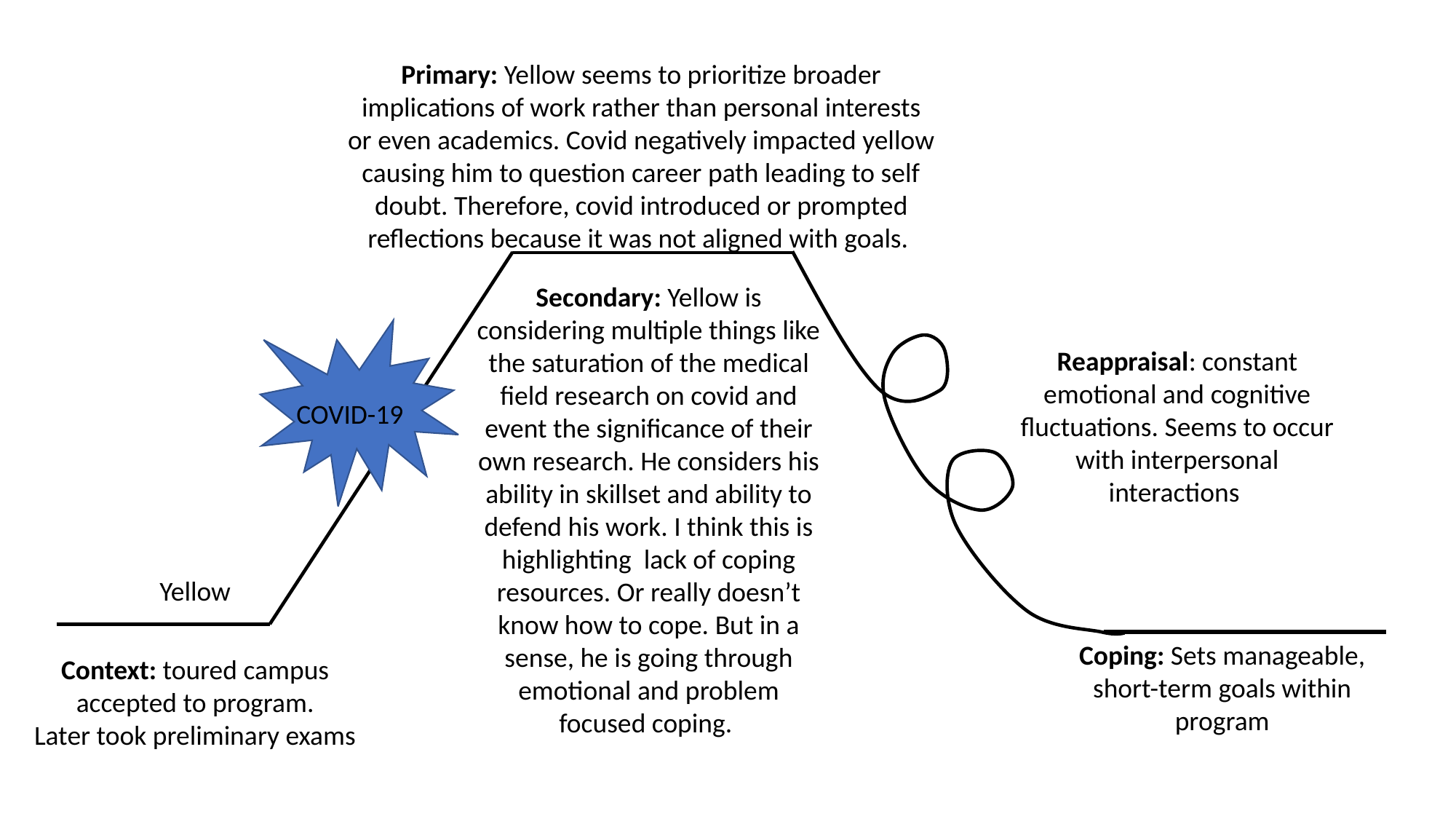

Primary: Yellow seems to prioritize broader implications of work rather than personal interests or even academics. Covid negatively impacted yellow causing him to question career path leading to self doubt. Therefore, covid introduced or prompted reflections because it was not aligned with goals.
Secondary: Yellow is considering multiple things like the saturation of the medical field research on covid and event the significance of their own research. He considers his ability in skillset and ability to defend his work. I think this is highlighting lack of coping resources. Or really doesn’t know how to cope. But in a sense, he is going through emotional and problem focused coping.
Reappraisal: constant emotional and cognitive fluctuations. Seems to occur with interpersonal interactions
COVID-19
Yellow
Coping: Sets manageable, short-term goals within program
Context: toured campus accepted to program.
Later took preliminary exams

## Slide 6
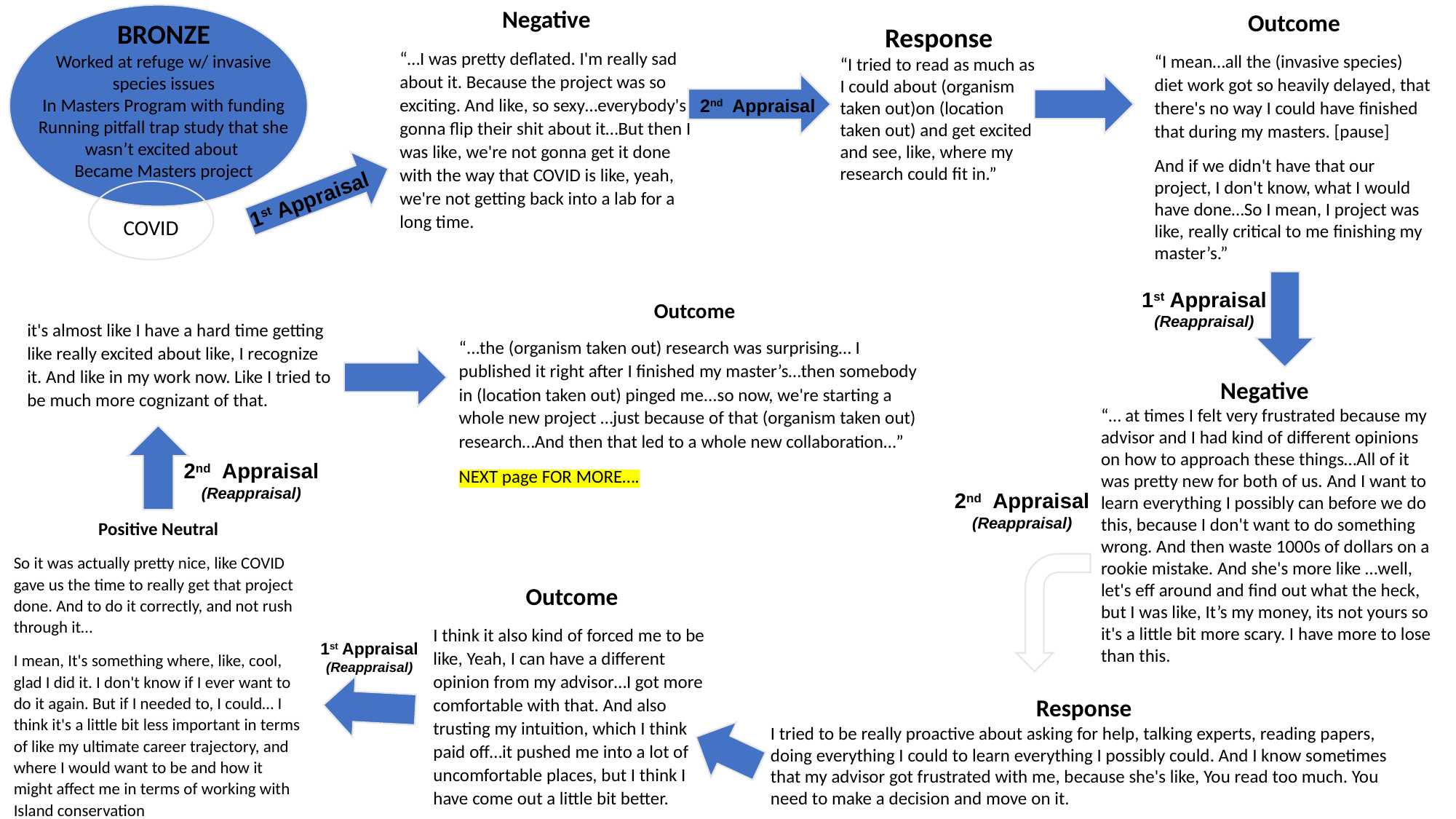

Outcome
“I mean…all the (invasive species) diet work got so heavily delayed, that there's no way I could have finished that during my masters. [pause]
And if we didn't have that our project, I don't know, what I would have done…So I mean, I project was like, really critical to me finishing my master’s.”
BRONZE
Worked at refuge w/ invasive species issues
In Masters Program with funding
Running pitfall trap study that she wasn’t excited about
Became Masters project
Negative
“…I was pretty deflated. I'm really sad about it. Because the project was so exciting. And like, so sexy…everybody's gonna flip their shit about it…But then I was like, we're not gonna get it done with the way that COVID is like, yeah, we're not getting back into a lab for a long time.
Response
“I tried to read as much as I could about (organism taken out)on (location taken out) and get excited and see, like, where my research could fit in.”
2nd Appraisal
1st Appraisal
COVID
1st Appraisal
(Reappraisal)
Outcome
“...the (organism taken out) research was surprising… I published it right after I finished my master’s…then somebody in (location taken out) pinged me...so now, we're starting a whole new project …just because of that (organism taken out) research…And then that led to a whole new collaboration…”
NEXT page FOR MORE….
it's almost like I have a hard time getting like really excited about like, I recognize it. And like in my work now. Like I tried to be much more cognizant of that.
Negative
“… at times I felt very frustrated because my advisor and I had kind of different opinions on how to approach these things…All of it was pretty new for both of us. And I want to learn everything I possibly can before we do this, because I don't want to do something wrong. And then waste 1000s of dollars on a rookie mistake. And she's more like …well, let's eff around and find out what the heck, but I was like, It’s my money, its not yours so it's a little bit more scary. I have more to lose than this.
2nd Appraisal
(Reappraisal)
2nd Appraisal
(Reappraisal)
Positive Neutral
So it was actually pretty nice, like COVID gave us the time to really get that project done. And to do it correctly, and not rush through it…
I mean, It's something where, like, cool, glad I did it. I don't know if I ever want to do it again. But if I needed to, I could… I think it's a little bit less important in terms of like my ultimate career trajectory, and where I would want to be and how it might affect me in terms of working with Island conservation
Outcome
I think it also kind of forced me to be like, Yeah, I can have a different opinion from my advisor…I got more comfortable with that. And also trusting my intuition, which I think paid off…it pushed me into a lot of uncomfortable places, but I think I have come out a little bit better.
1st Appraisal
(Reappraisal)
Response
I tried to be really proactive about asking for help, talking experts, reading papers, doing everything I could to learn everything I possibly could. And I know sometimes that my advisor got frustrated with me, because she's like, You read too much. You need to make a decision and move on it.

## Slide 7
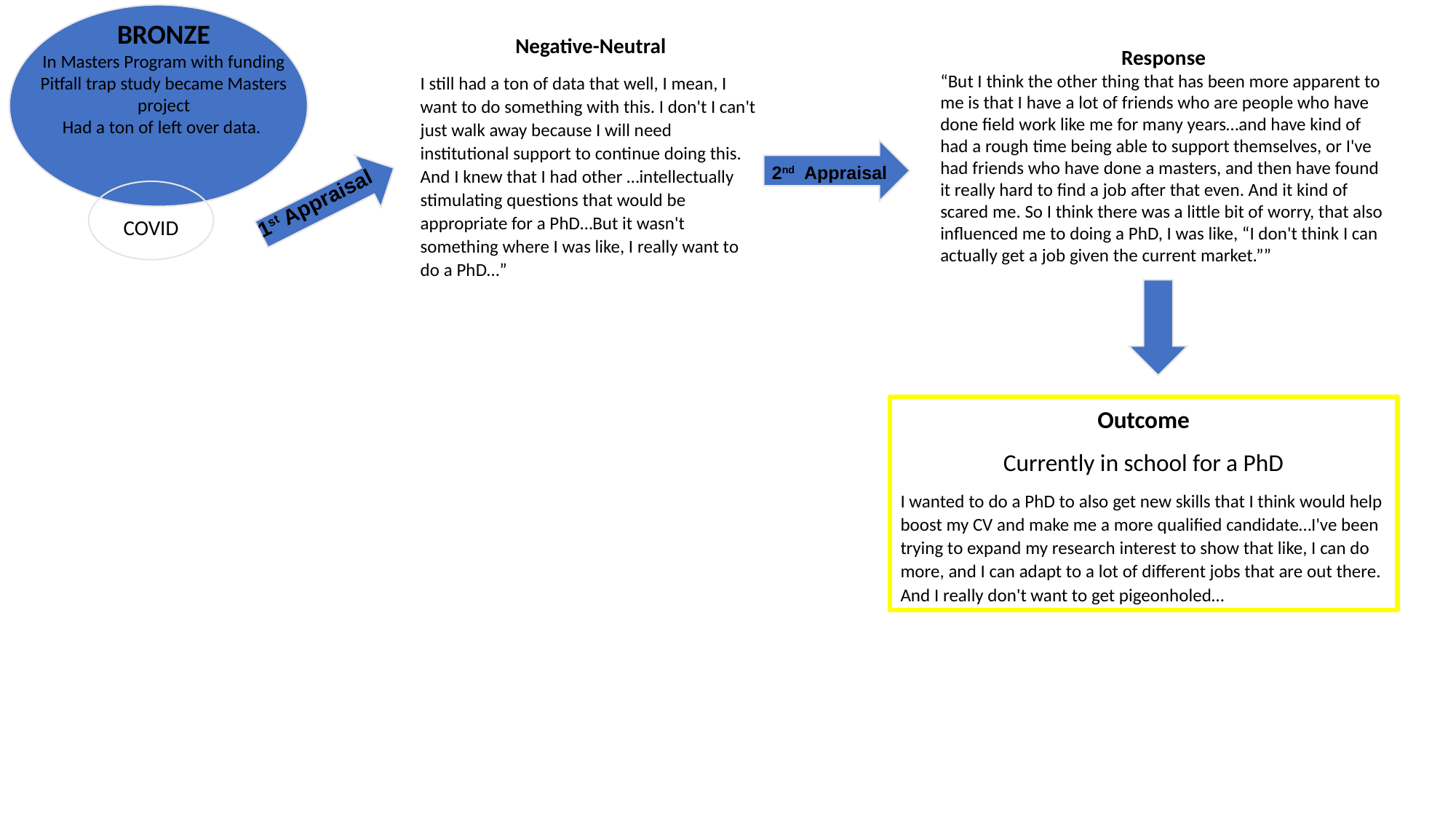

BRONZE
In Masters Program with funding
Pitfall trap study became Masters project
Had a ton of left over data.
Negative-Neutral
I still had a ton of data that well, I mean, I want to do something with this. I don't I can't just walk away because I will need institutional support to continue doing this. And I knew that I had other …intellectually stimulating questions that would be appropriate for a PhD…But it wasn't something where I was like, I really want to do a PhD…”
Response
“But I think the other thing that has been more apparent to me is that I have a lot of friends who are people who have done field work like me for many years…and have kind of had a rough time being able to support themselves, or I've had friends who have done a masters, and then have found it really hard to find a job after that even. And it kind of scared me. So I think there was a little bit of worry, that also influenced me to doing a PhD, I was like, “I don't think I can actually get a job given the current market.””
2nd Appraisal
1st Appraisal
COVID
Outcome
Currently in school for a PhD
I wanted to do a PhD to also get new skills that I think would help boost my CV and make me a more qualified candidate…I've been trying to expand my research interest to show that like, I can do more, and I can adapt to a lot of different jobs that are out there. And I really don't want to get pigeonholed…

## Slide 8
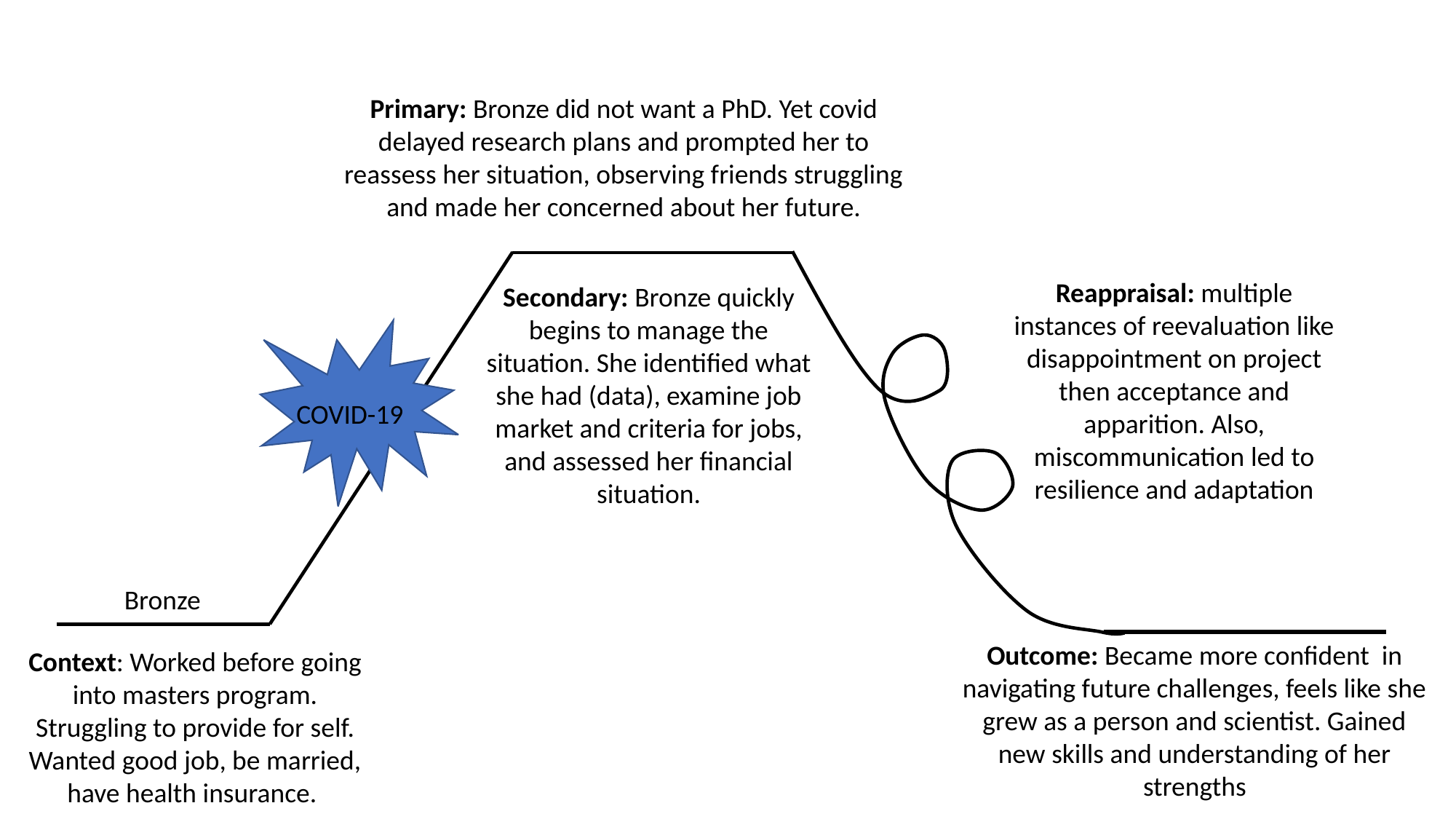

Primary: Bronze did not want a PhD. Yet covid delayed research plans and prompted her to reassess her situation, observing friends struggling and made her concerned about her future.
Reappraisal: multiple instances of reevaluation like disappointment on project then acceptance and apparition. Also, miscommunication led to resilience and adaptation
Secondary: Bronze quickly begins to manage the situation. She identified what she had (data), examine job market and criteria for jobs, and assessed her financial situation.
COVID-19
Bronze
Outcome: Became more confident in navigating future challenges, feels like she grew as a person and scientist. Gained new skills and understanding of her strengths
Context: Worked before going into masters program. Struggling to provide for self. Wanted good job, be married, have health insurance.

## Slide 9
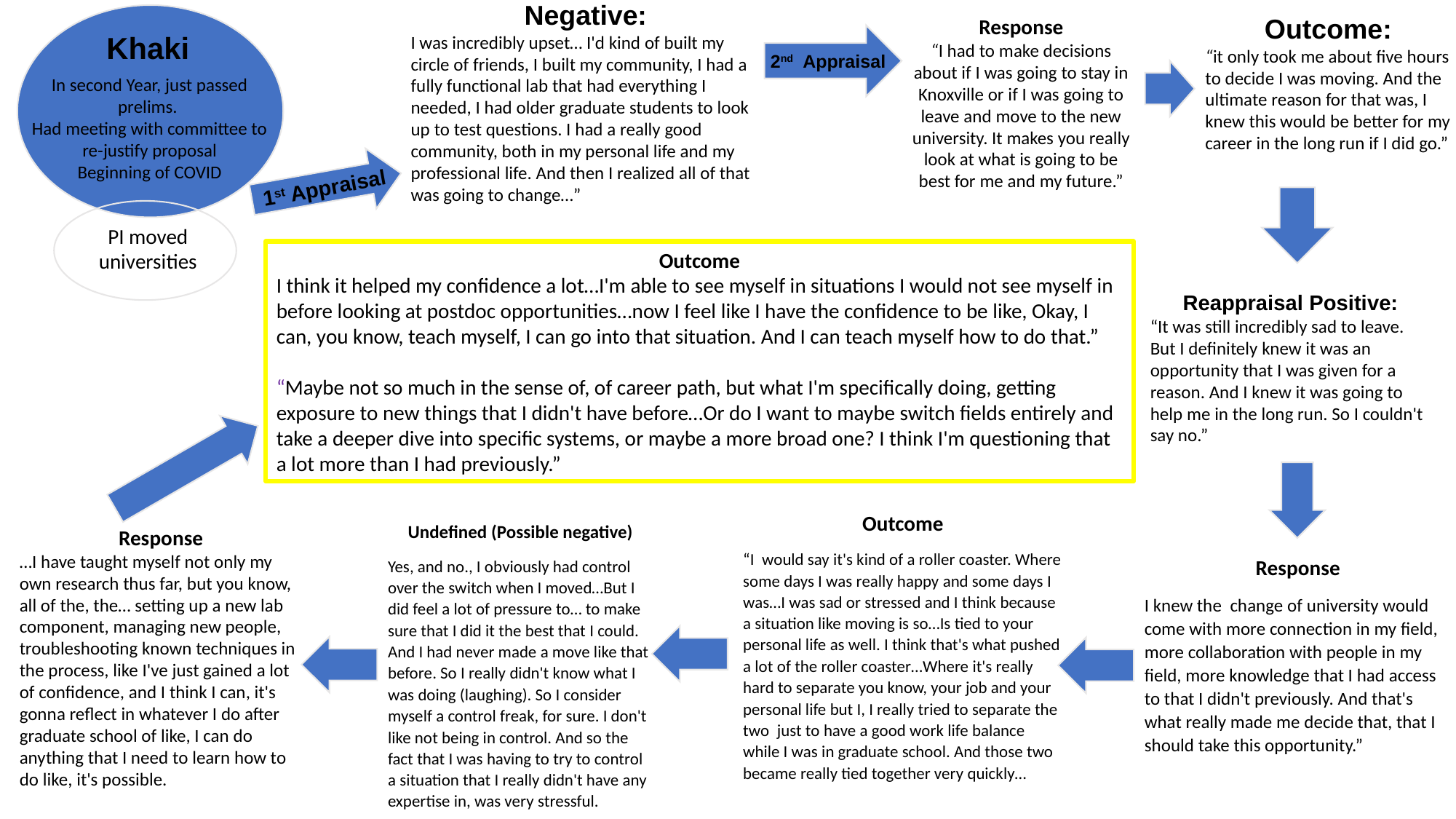

Negative:
I was incredibly upset… I'd kind of built my circle of friends, I built my community, I had a fully functional lab that had everything I needed, I had older graduate students to look up to test questions. I had a really good community, both in my personal life and my professional life. And then I realized all of that was going to change…”
Outcome:
“it only took me about five hours to decide I was moving. And the ultimate reason for that was, I knew this would be better for my career in the long run if I did go.”
Response
“I had to make decisions about if I was going to stay in Knoxville or if I was going to leave and move to the new university. It makes you really look at what is going to be best for me and my future.”
Khaki
2nd Appraisal
In second Year, just passed prelims.
Had meeting with committee to re-justify proposal
Beginning of COVID
1st Appraisal
PI moved universities
Outcome
I think it helped my confidence a lot…I'm able to see myself in situations I would not see myself in before looking at postdoc opportunities…now I feel like I have the confidence to be like, Okay, I can, you know, teach myself, I can go into that situation. And I can teach myself how to do that.”
“Maybe not so much in the sense of, of career path, but what I'm specifically doing, getting exposure to new things that I didn't have before…Or do I want to maybe switch fields entirely and take a deeper dive into specific systems, or maybe a more broad one? I think I'm questioning that a lot more than I had previously.”
Reappraisal Positive:
“It was still incredibly sad to leave. But I definitely knew it was an opportunity that I was given for a reason. And I knew it was going to help me in the long run. So I couldn't say no.”
Outcome
“I  would say it's kind of a roller coaster. Where some days I was really happy and some days I was…I was sad or stressed and I think because a situation like moving is so…Is tied to your personal life as well. I think that's what pushed a lot of the roller coaster…Where it's really hard to separate you know, your job and your personal life but I, I really tried to separate the two  just to have a good work life balance while I was in graduate school. And those two became really tied together very quickly…
Undefined (Possible negative)
Yes, and no., I obviously had control over the switch when I moved…But I did feel a lot of pressure to… to make sure that I did it the best that I could. And I had never made a move like that before. So I really didn't know what I was doing (laughing). So I consider myself a control freak, for sure. I don't like not being in control. And so the fact that I was having to try to control a situation that I really didn't have any expertise in, was very stressful.
Response
…I have taught myself not only my own research thus far, but you know, all of the, the… setting up a new lab component, managing new people, troubleshooting known techniques in the process, like I've just gained a lot of confidence, and I think I can, it's gonna reflect in whatever I do after graduate school of like, I can do anything that I need to learn how to do like, it's possible.
Response
I knew the  change of university would come with more connection in my field, more collaboration with people in my field, more knowledge that I had access to that I didn't previously. And that's what really made me decide that, that I should take this opportunity.”

## Slide 10
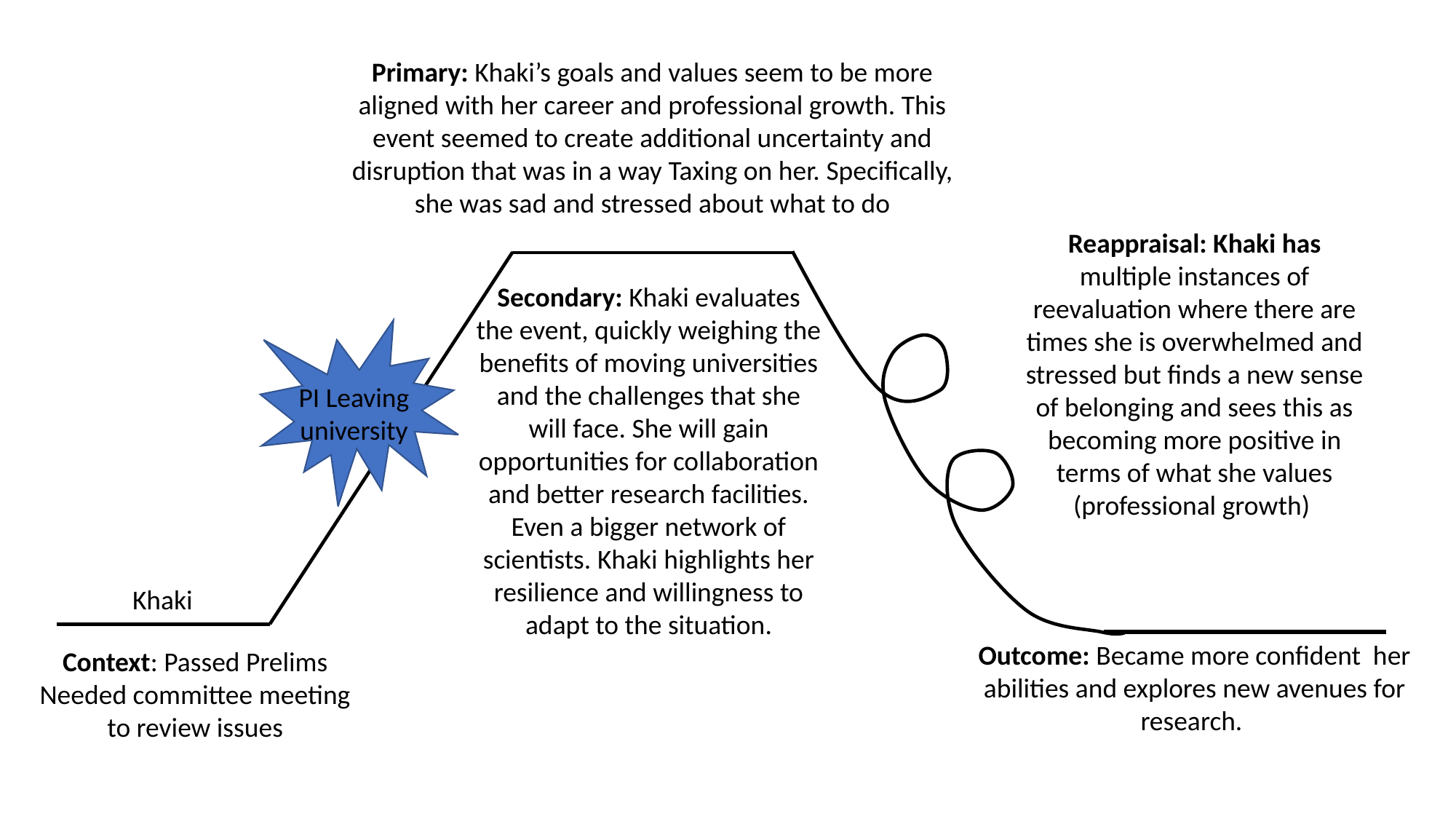

Primary: Khaki’s goals and values seem to be more aligned with her career and professional growth. This event seemed to create additional uncertainty and disruption that was in a way Taxing on her. Specifically, she was sad and stressed about what to do
Reappraisal: Khaki has multiple instances of reevaluation where there are times she is overwhelmed and stressed but finds a new sense of belonging and sees this as becoming more positive in terms of what she values (professional growth)
Secondary: Khaki evaluates the event, quickly weighing the benefits of moving universities and the challenges that she will face. She will gain opportunities for collaboration and better research facilities. Even a bigger network of scientists. Khaki highlights her resilience and willingness to adapt to the situation.
PI Leaving university
Khaki
Outcome: Became more confident her abilities and explores new avenues for research.
Context: Passed Prelims
Needed committee meeting to review issues

## Slide 11
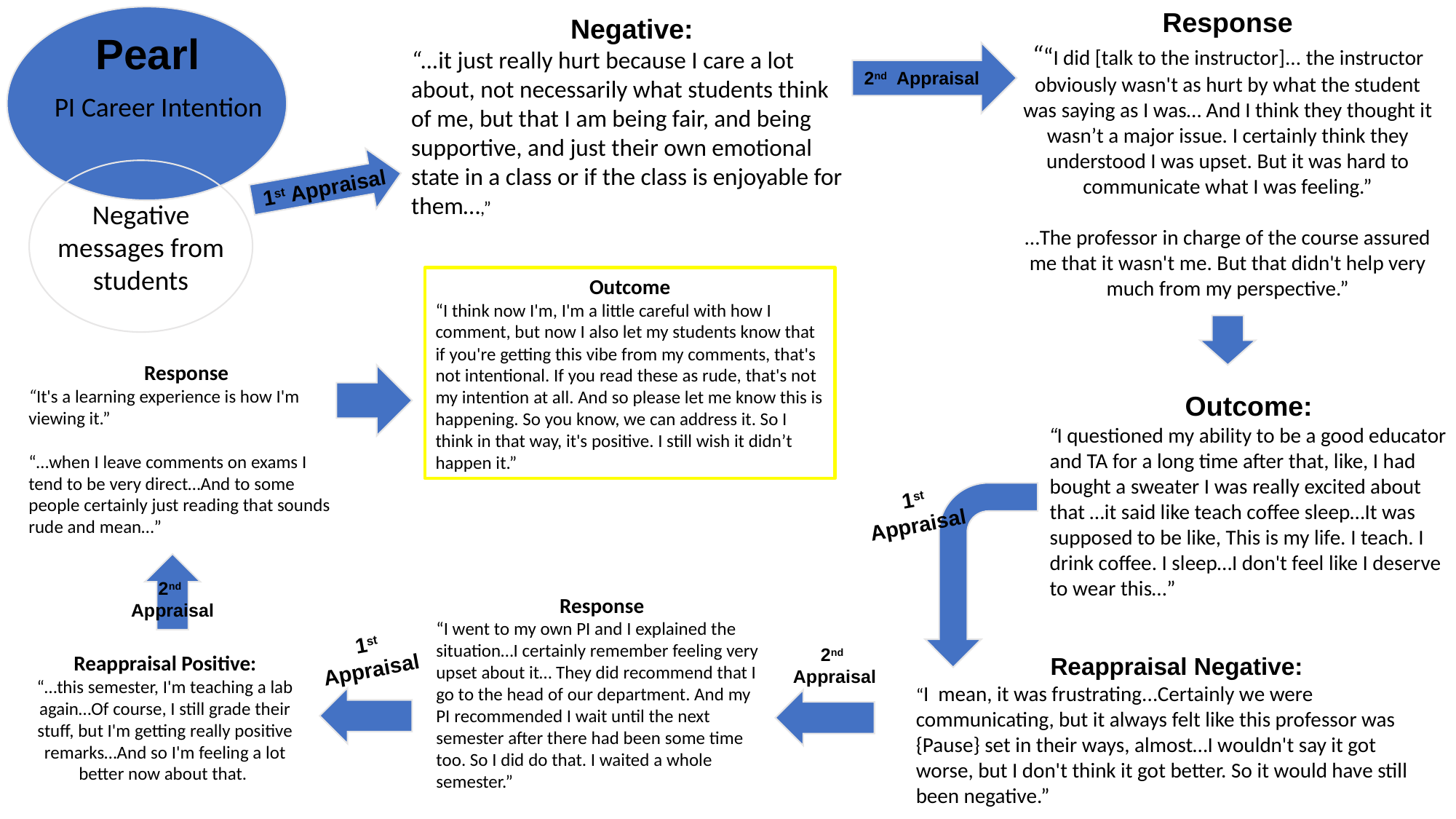

Response
““I did [talk to the instructor]... the instructor obviously wasn't as hurt by what the student was saying as I was… And I think they thought it wasn’t a major issue. I certainly think they understood I was upset. But it was hard to communicate what I was feeling.”
…The professor in charge of the course assured me that it wasn't me. But that didn't help very much from my perspective.”
Negative:
“…it just really hurt because I care a lot about, not necessarily what students think of me, but that I am being fair, and being supportive, and just their own emotional state in a class or if the class is enjoyable for them…,”
Pearl
2nd Appraisal
PI Career Intention
1st Appraisal
Negative messages from students
Outcome
“I think now I'm, I'm a little careful with how I comment, but now I also let my students know that if you're getting this vibe from my comments, that's not intentional. If you read these as rude, that's not my intention at all. And so please let me know this is happening. So you know, we can address it. So I think in that way, it's positive. I still wish it didn’t happen it.”
Response
“It's a learning experience is how I'm viewing it.”
“…when I leave comments on exams I tend to be very direct…And to some people certainly just reading that sounds rude and mean…”
Outcome:
“I questioned my ability to be a good educator and TA for a long time after that, like, I had bought a sweater I was really excited about that …it said like teach coffee sleep…It was supposed to be like, This is my life. I teach. I drink coffee. I sleep…I don't feel like I deserve to wear this…”
1st Appraisal
2nd Appraisal
Response
“I went to my own PI and I explained the situation…I certainly remember feeling very upset about it… They did recommend that I go to the head of our department. And my PI recommended I wait until the next semester after there had been some time too. So I did do that. I waited a whole semester.”
1st Appraisal
2nd Appraisal
Reappraisal Positive:
“…this semester, I'm teaching a lab again…Of course, I still grade their stuff, but I'm getting really positive remarks…And so I'm feeling a lot better now about that.
Reappraisal Negative:
“I  mean, it was frustrating...Certainly we were communicating, but it always felt like this professor was {Pause} set in their ways, almost…I wouldn't say it got worse, but I don't think it got better. So it would have still been negative.”

## Slide 12
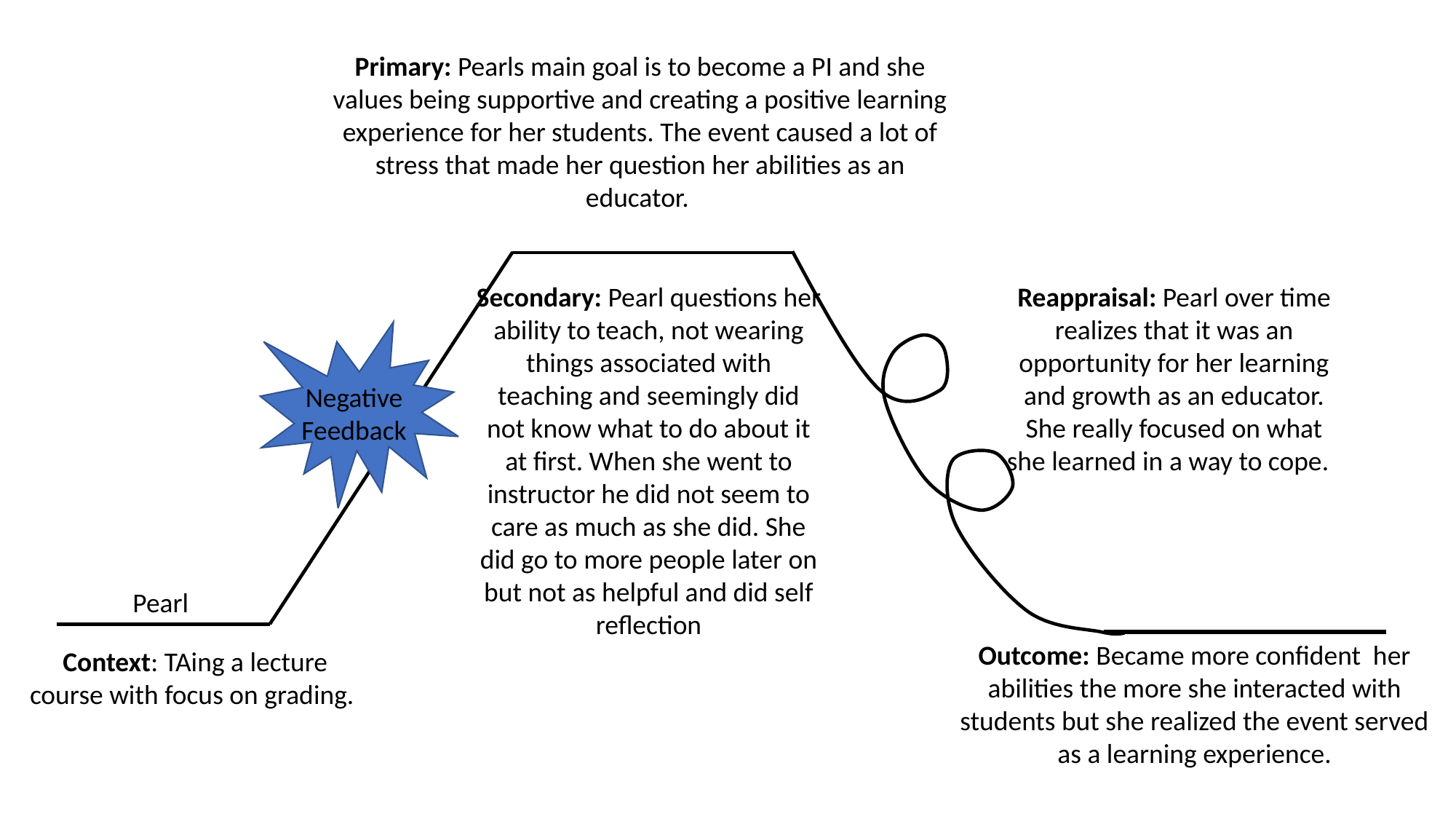

Primary: Pearls main goal is to become a PI and she values being supportive and creating a positive learning experience for her students. The event caused a lot of stress that made her question her abilities as an educator.
Secondary: Pearl questions her ability to teach, not wearing things associated with teaching and seemingly did not know what to do about it at first. When she went to instructor he did not seem to care as much as she did. She did go to more people later on but not as helpful and did self reflection
Reappraisal: Pearl over time realizes that it was an opportunity for her learning and growth as an educator. She really focused on what she learned in a way to cope.
Negative Feedback
Pearl
Outcome: Became more confident her abilities the more she interacted with students but she realized the event served as a learning experience.
Context: TAing a lecture course with focus on grading.

## Slide 13
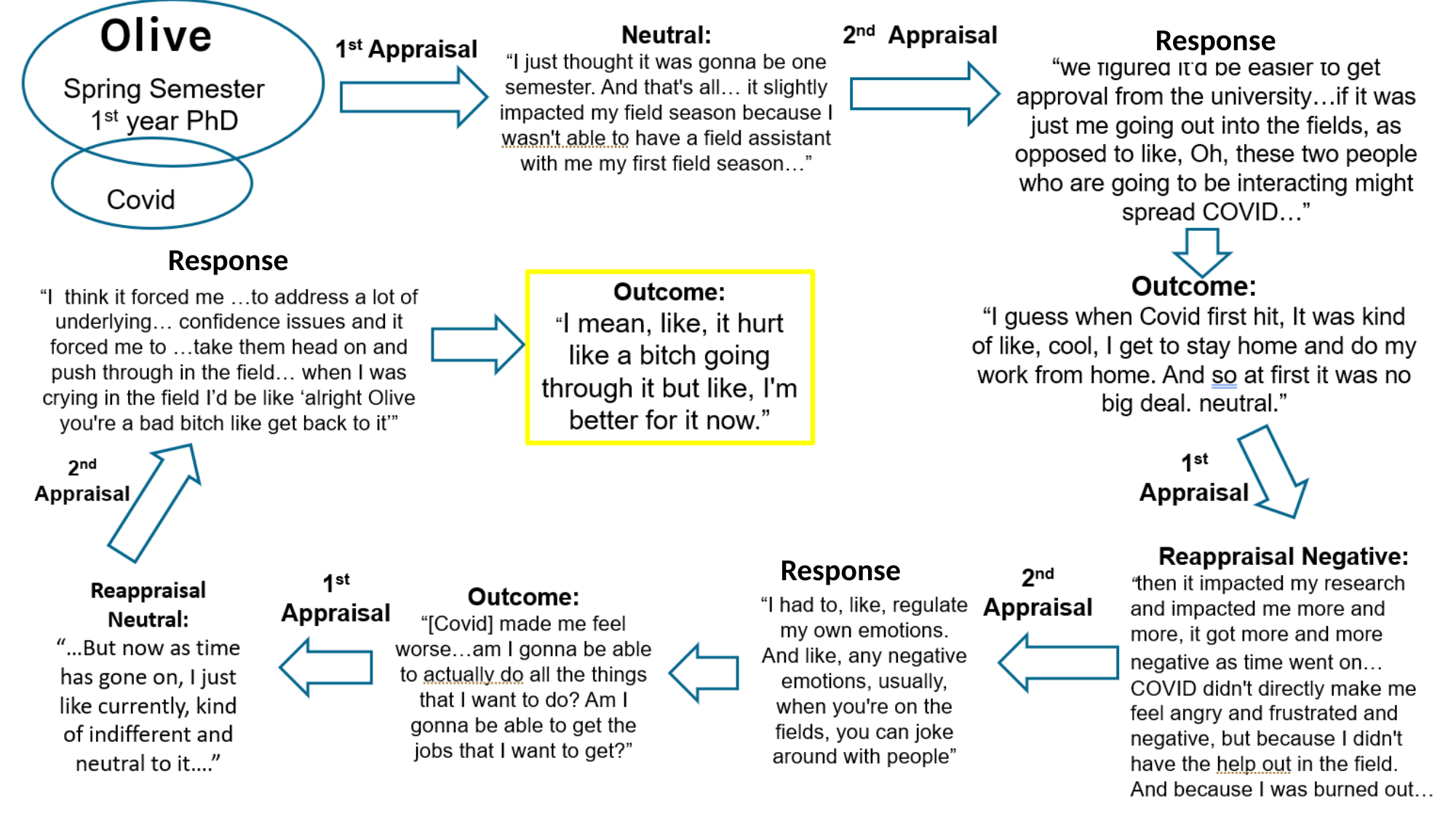

Response
Response
Response

## Slide 14
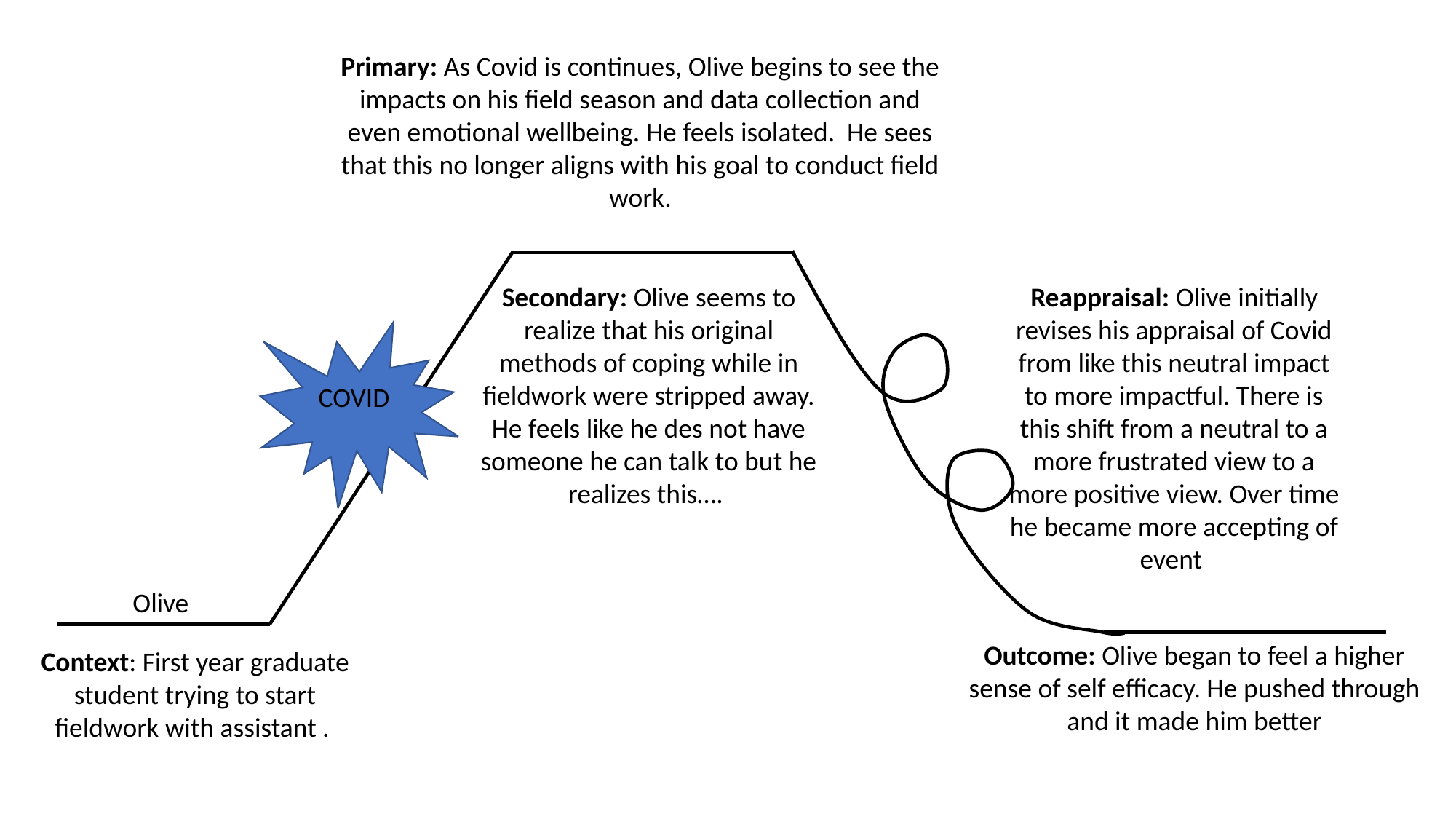

Primary: As Covid is continues, Olive begins to see the impacts on his field season and data collection and even emotional wellbeing. He feels isolated. He sees that this no longer aligns with his goal to conduct field work.
Secondary: Olive seems to realize that his original methods of coping while in fieldwork were stripped away. He feels like he des not have someone he can talk to but he realizes this….
Reappraisal: Olive initially revises his appraisal of Covid from like this neutral impact to more impactful. There is this shift from a neutral to a more frustrated view to a more positive view. Over time he became more accepting of event
COVID
Olive
Outcome: Olive began to feel a higher sense of self efficacy. He pushed through and it made him better
Context: First year graduate student trying to start fieldwork with assistant .

## Slide 15
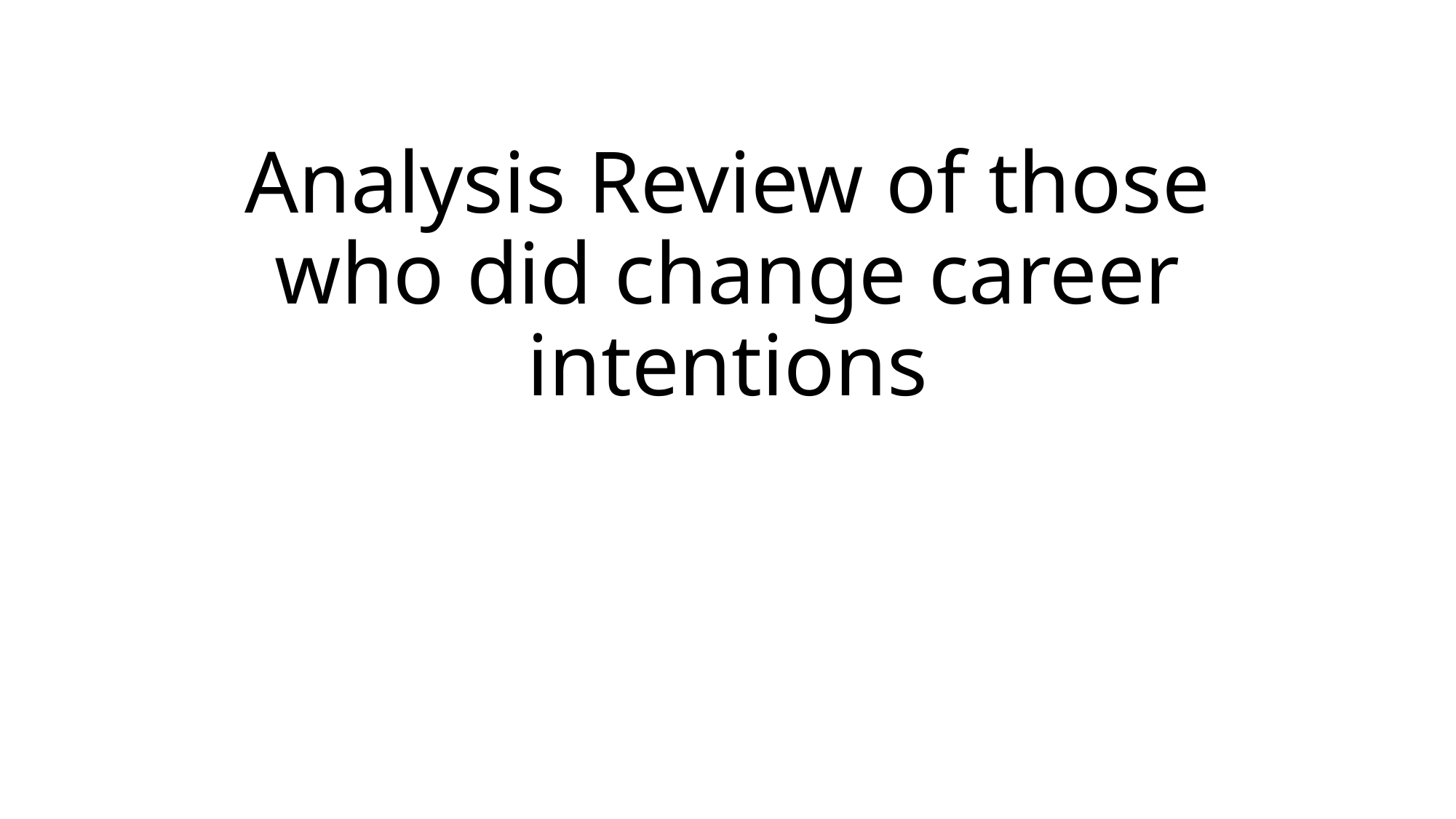

# Analysis Review of those who did change career intentions

## Slide 16
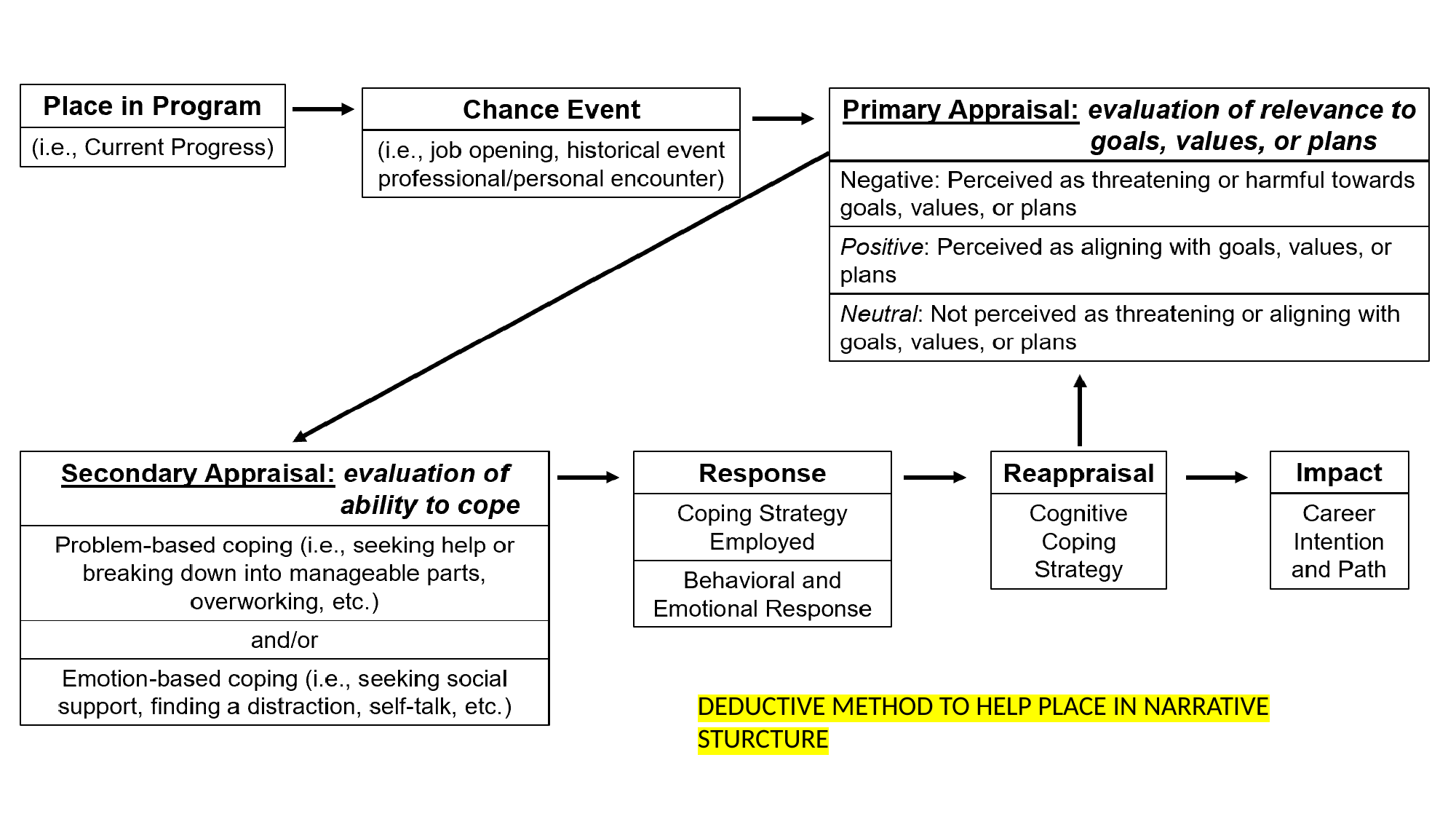

DEDUCTIVE METHOD TO HELP PLACE IN NARRATIVE STURCTURE

## Slide 17
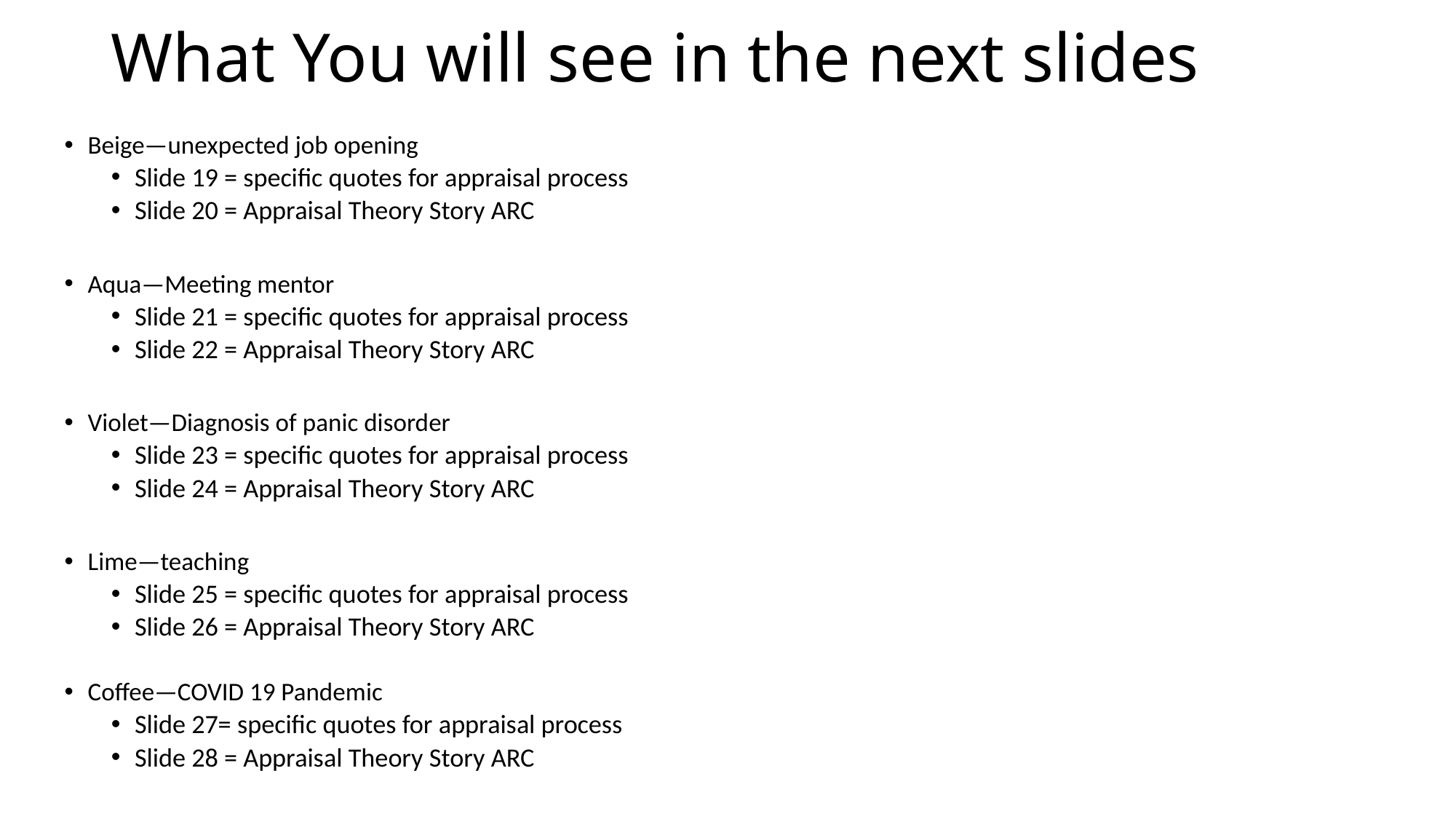

# What You will see in the next slides
Beige—unexpected job opening
Slide 19 = specific quotes for appraisal process
Slide 20 = Appraisal Theory Story ARC
Aqua—Meeting mentor
Slide 21 = specific quotes for appraisal process
Slide 22 = Appraisal Theory Story ARC
Violet—Diagnosis of panic disorder
Slide 23 = specific quotes for appraisal process
Slide 24 = Appraisal Theory Story ARC
Lime—teaching
Slide 25 = specific quotes for appraisal process
Slide 26 = Appraisal Theory Story ARC
Coffee—COVID 19 Pandemic
Slide 27= specific quotes for appraisal process
Slide 28 = Appraisal Theory Story ARC

## Slide 18
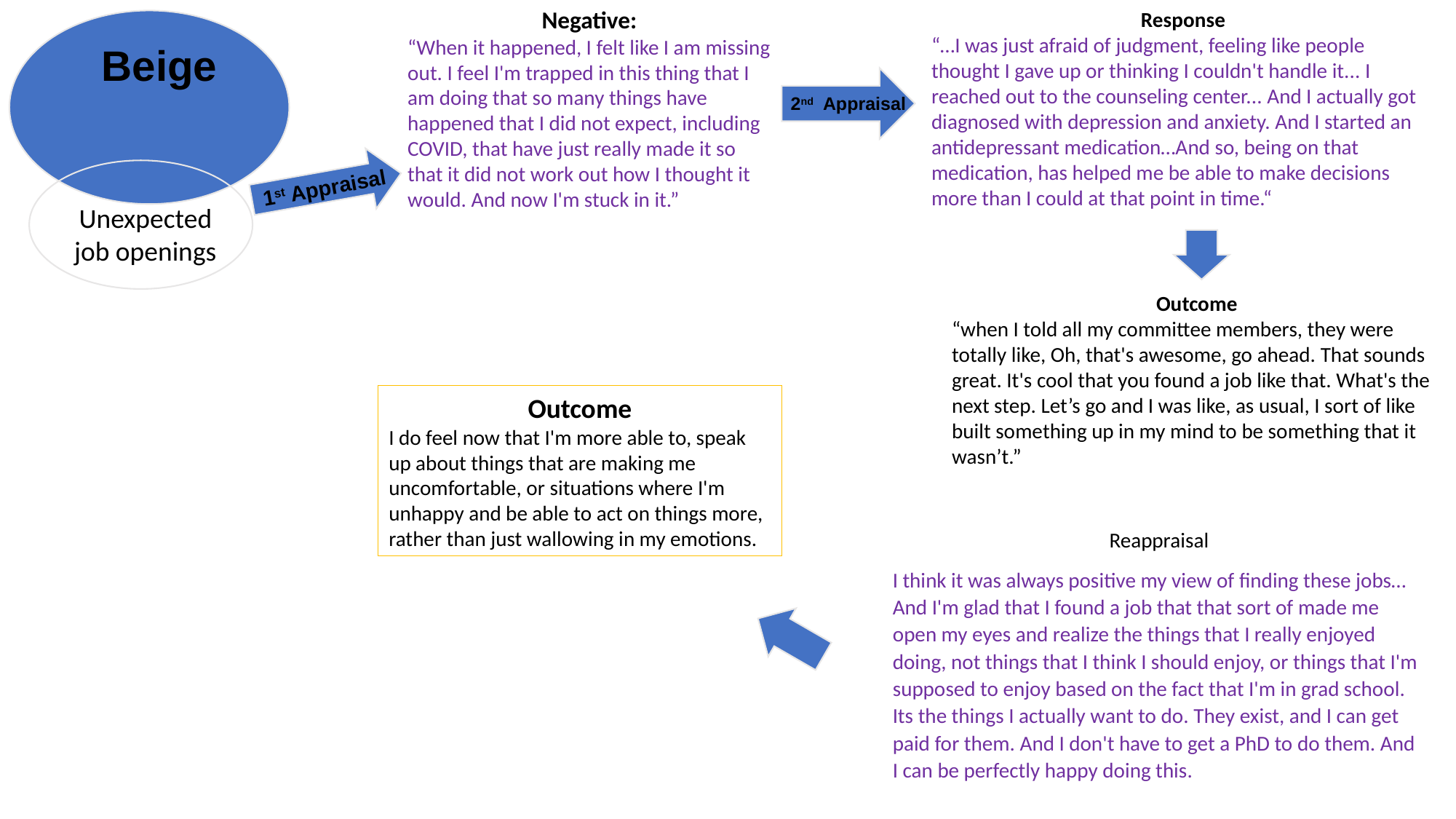

Negative:
“When it happened, I felt like I am missing out. I feel I'm trapped in this thing that I am doing that so many things have happened that I did not expect, including COVID, that have just really made it so that it did not work out how I thought it would. And now I'm stuck in it.”
Response
“…I was just afraid of judgment, feeling like people thought I gave up or thinking I couldn't handle it... I reached out to the counseling center... And I actually got diagnosed with depression and anxiety. And I started an antidepressant medication…And so, being on that medication, has helped me be able to make decisions more than I could at that point in time.“
Beige
2nd Appraisal
1st Appraisal
Unexpected job openings
Outcome
“when I told all my committee members, they were totally like, Oh, that's awesome, go ahead. That sounds great. It's cool that you found a job like that. What's the next step. Let’s go and I was like, as usual, I sort of like built something up in my mind to be something that it wasn’t.”
Outcome
I do feel now that I'm more able to, speak up about things that are making me uncomfortable, or situations where I'm unhappy and be able to act on things more, rather than just wallowing in my emotions.
Reappraisal
I think it was always positive my view of finding these jobs…And I'm glad that I found a job that that sort of made me open my eyes and realize the things that I really enjoyed doing, not things that I think I should enjoy, or things that I'm supposed to enjoy based on the fact that I'm in grad school. Its the things I actually want to do. They exist, and I can get paid for them. And I don't have to get a PhD to do them. And I can be perfectly happy doing this.

## Slide 19
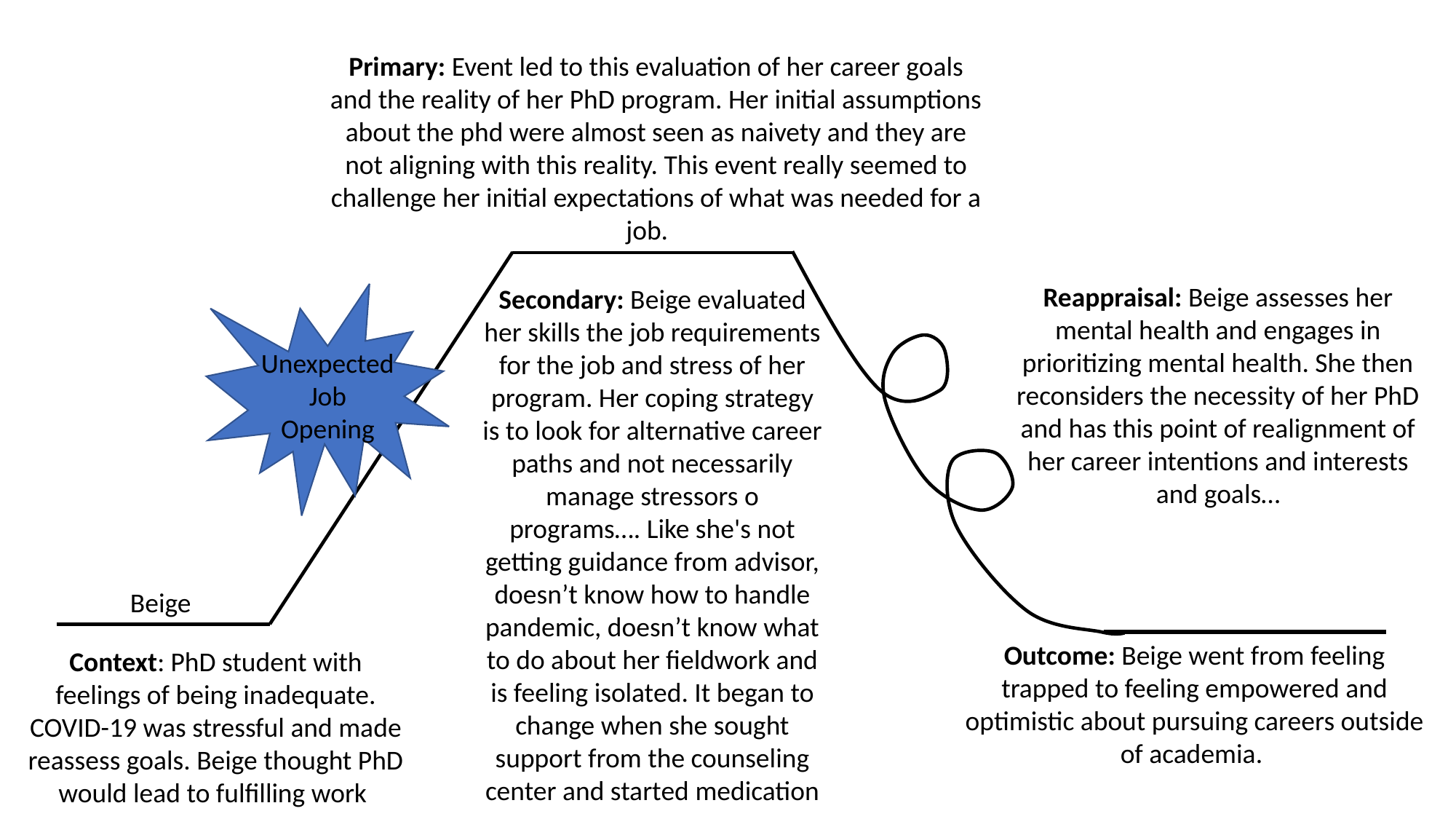

Primary: Event led to this evaluation of her career goals and the reality of her PhD program. Her initial assumptions about the phd were almost seen as naivety and they are not aligning with this reality. This event really seemed to challenge her initial expectations of what was needed for a job.
Reappraisal: Beige assesses her mental health and engages in prioritizing mental health. She then reconsiders the necessity of her PhD and has this point of realignment of her career intentions and interests and goals…
Secondary: Beige evaluated her skills the job requirements for the job and stress of her program. Her coping strategy is to look for alternative career paths and not necessarily manage stressors o programs…. Like she's not getting guidance from advisor, doesn’t know how to handle pandemic, doesn’t know what to do about her fieldwork and is feeling isolated. It began to change when she sought support from the counseling center and started medication
Unexpected Job Opening
Beige
Outcome: Beige went from feeling trapped to feeling empowered and optimistic about pursuing careers outside of academia.
Context: PhD student with feelings of being inadequate. COVID-19 was stressful and made reassess goals. Beige thought PhD would lead to fulfilling work

## Slide 20
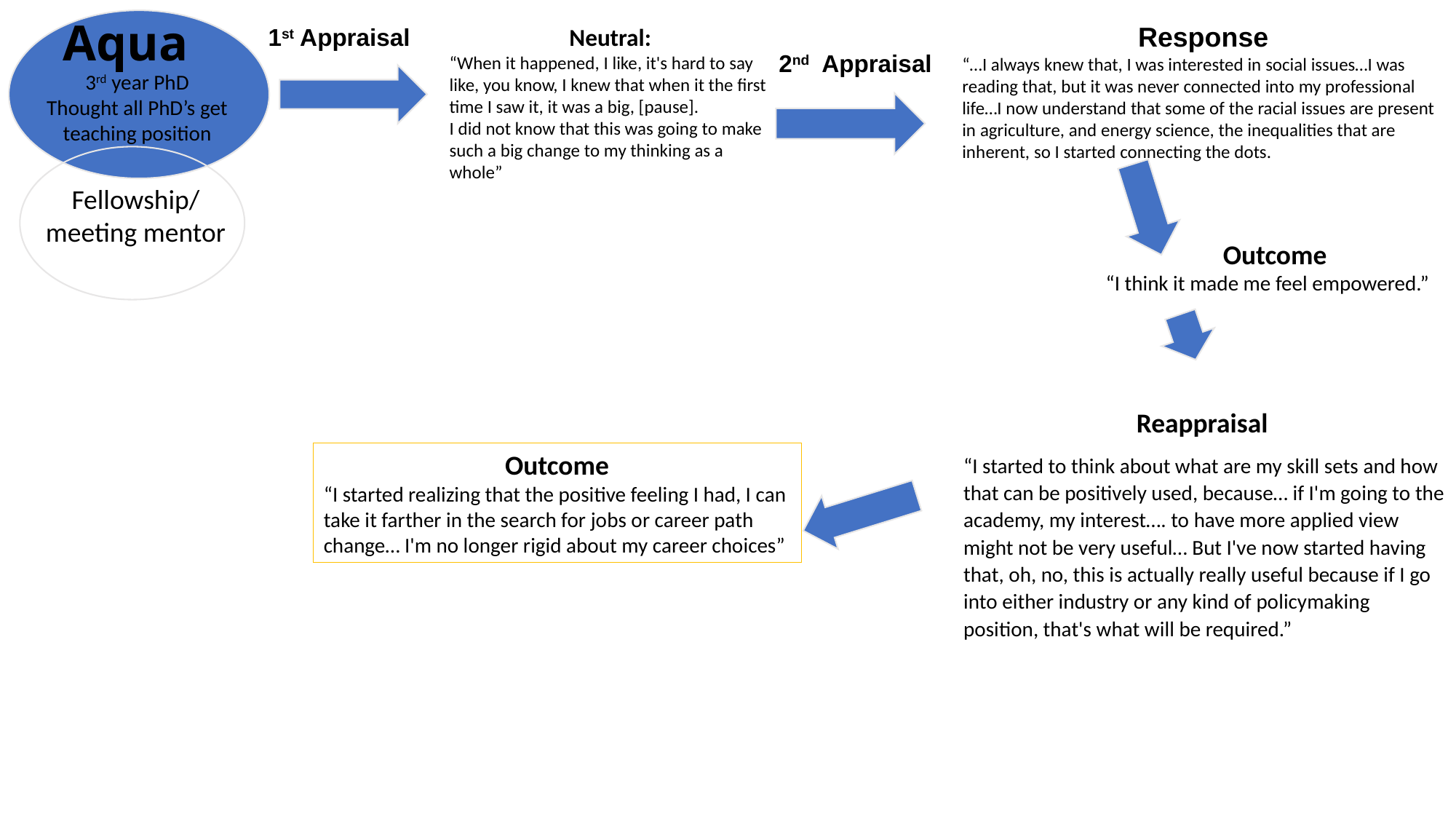

Aqua
Response
“…I always knew that, I was interested in social issues…I was reading that, but it was never connected into my professional life…I now understand that some of the racial issues are present in agriculture, and energy science, the inequalities that are inherent, so I started connecting the dots.
1st Appraisal
Neutral:
“When it happened, I like, it's hard to say like, you know, I knew that when it the first time I saw it, it was a big, [pause].
I did not know that this was going to make such a big change to my thinking as a whole”
2nd Appraisal
3rd year PhD
Thought all PhD’s get teaching position
Fellowship/ meeting mentor
Outcome
“I think it made me feel empowered.”
Reappraisal
“I started to think about what are my skill sets and how that can be positively used, because… if I'm going to the academy, my interest…. to have more applied view might not be very useful… But I've now started having that, oh, no, this is actually really useful because if I go into either industry or any kind of policymaking position, that's what will be required.”
Outcome
“I started realizing that the positive feeling I had, I can take it farther in the search for jobs or career path change… I'm no longer rigid about my career choices”

## Slide 21
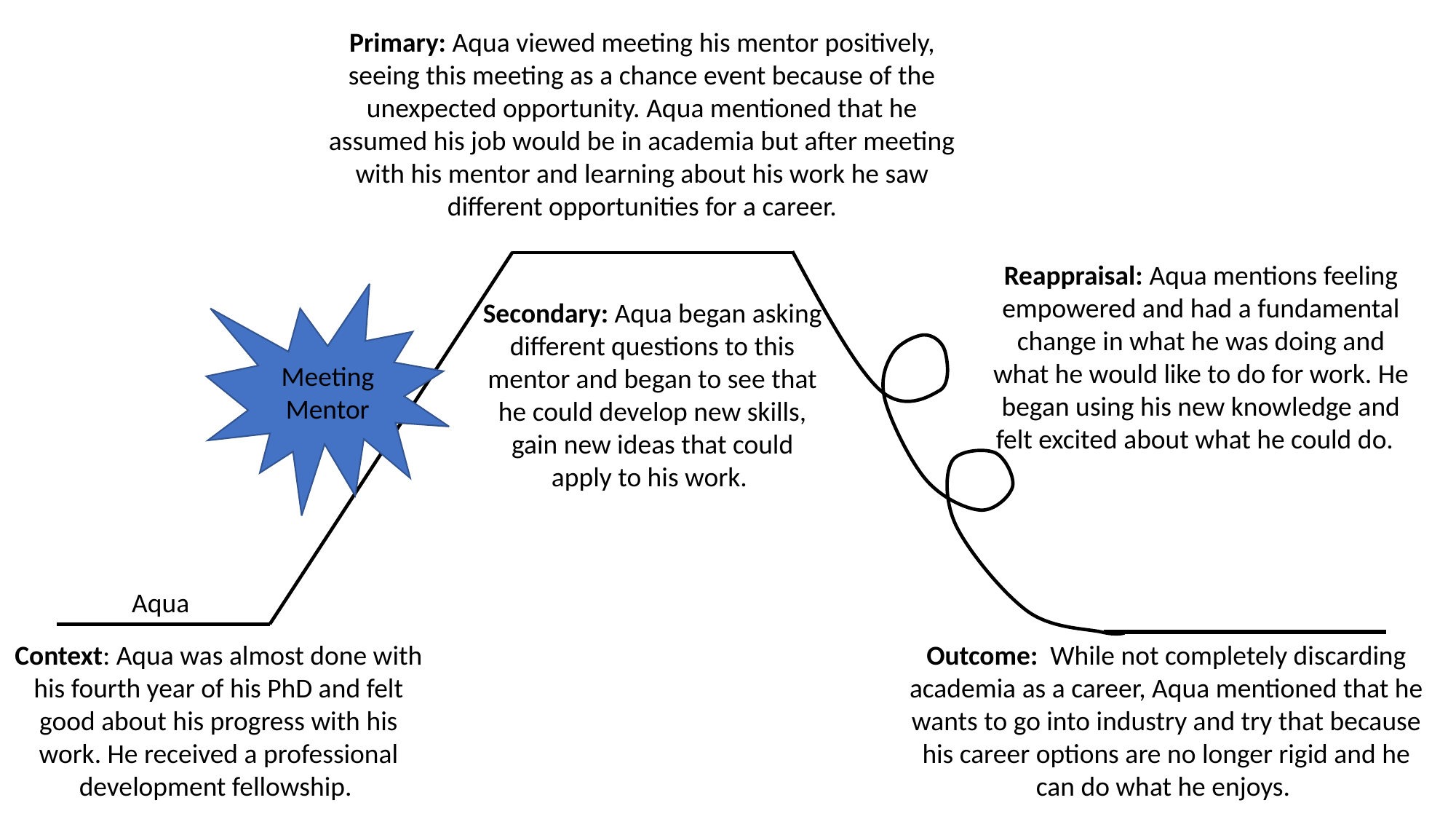

Primary: Aqua viewed meeting his mentor positively, seeing this meeting as a chance event because of the unexpected opportunity. Aqua mentioned that he assumed his job would be in academia but after meeting with his mentor and learning about his work he saw different opportunities for a career.
Reappraisal: Aqua mentions feeling empowered and had a fundamental change in what he was doing and what he would like to do for work. He began using his new knowledge and felt excited about what he could do.
Secondary: Aqua began asking different questions to this mentor and began to see that he could develop new skills, gain new ideas that could apply to his work.
Meeting Mentor
Aqua
Context: Aqua was almost done with his fourth year of his PhD and felt good about his progress with his work. He received a professional development fellowship.
Outcome: While not completely discarding academia as a career, Aqua mentioned that he wants to go into industry and try that because his career options are no longer rigid and he can do what he enjoys.

## Slide 22
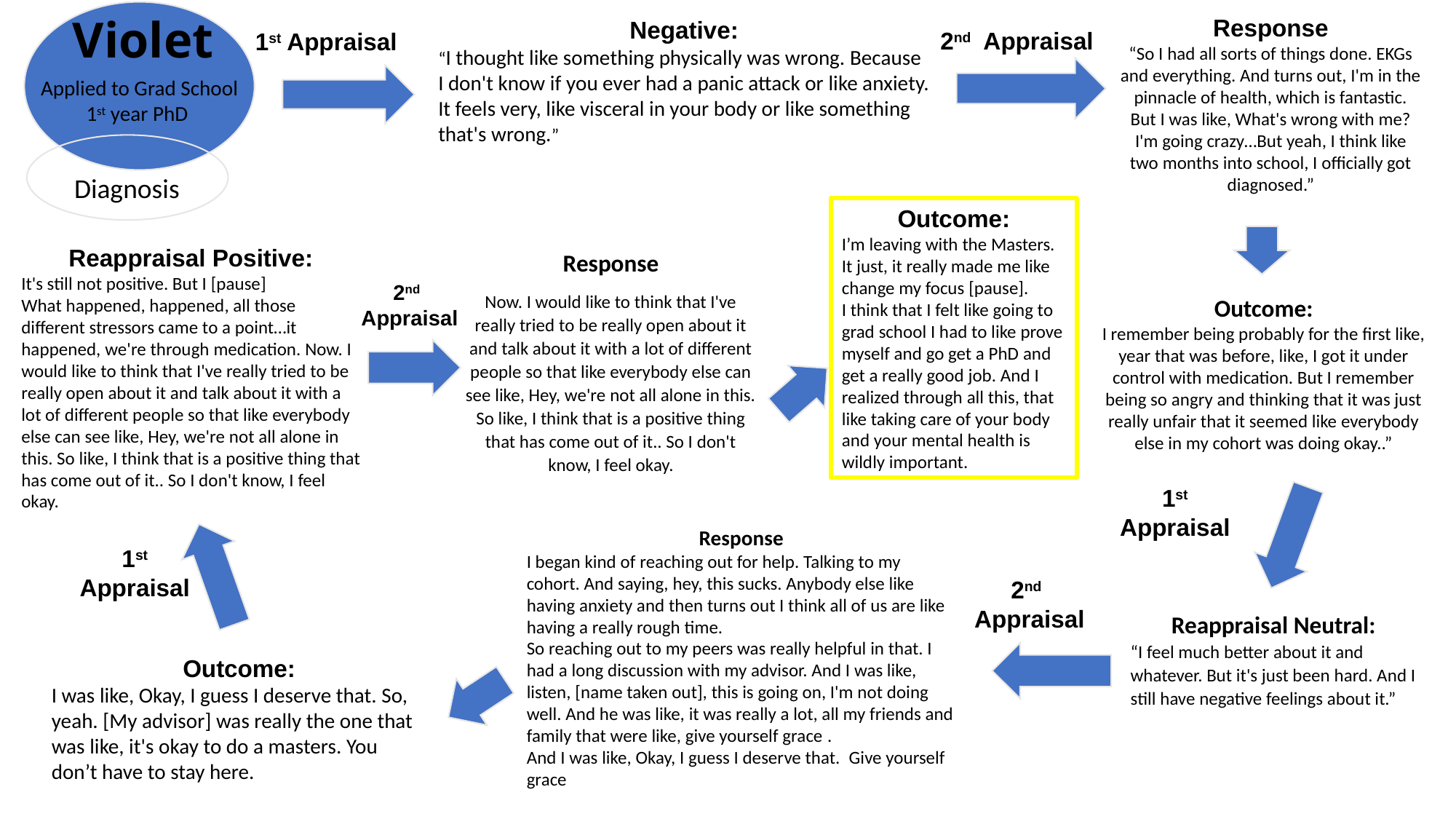

Violet
Response
“So I had all sorts of things done. EKGs and everything. And turns out, I'm in the pinnacle of health, which is fantastic. But I was like, What's wrong with me? I'm going crazy…But yeah, I think like two months into school, I officially got diagnosed.”
Negative:
“I thought like something physically was wrong. Because I don't know if you ever had a panic attack or like anxiety. It feels very, like visceral in your body or like something that's wrong.”
2nd Appraisal
1st Appraisal
Applied to Grad School
1st year PhD
Diagnosis
Outcome:
I’m leaving with the Masters. It just, it really made me like change my focus [pause].
I think that I felt like going to grad school I had to like prove myself and go get a PhD and get a really good job. And I realized through all this, that like taking care of your body and your mental health is wildly important.
Reappraisal Positive:
It's still not positive. But I [pause]
What happened, happened, all those different stressors came to a point…it happened, we're through medication. Now. I would like to think that I've really tried to be really open about it and talk about it with a lot of different people so that like everybody else can see like, Hey, we're not all alone in this. So like, I think that is a positive thing that has come out of it.. So I don't know, I feel okay.
Response
Now. I would like to think that I've really tried to be really open about it and talk about it with a lot of different people so that like everybody else can see like, Hey, we're not all alone in this. So like, I think that is a positive thing that has come out of it.. So I don't know, I feel okay.
2nd Appraisal
Outcome:
I remember being probably for the first like, year that was before, like, I got it under control with medication. But I remember being so angry and thinking that it was just really unfair that it seemed like everybody else in my cohort was doing okay..”
1st Appraisal
Response
I began kind of reaching out for help. Talking to my cohort. And saying, hey, this sucks. Anybody else like having anxiety and then turns out I think all of us are like having a really rough time.
So reaching out to my peers was really helpful in that. I had a long discussion with my advisor. And I was like, listen, [name taken out], this is going on, I'm not doing well. And he was like, it was really a lot, all my friends and family that were like, give yourself grace .
And I was like, Okay, I guess I deserve that.  Give yourself grace
1st Appraisal
2nd Appraisal
Reappraisal Neutral:
“I feel much better about it and whatever. But it's just been hard. And I still have negative feelings about it.”
Outcome:
I was like, Okay, I guess I deserve that. So, yeah. [My advisor] was really the one that was like, it's okay to do a masters. You don’t have to stay here.

## Slide 23
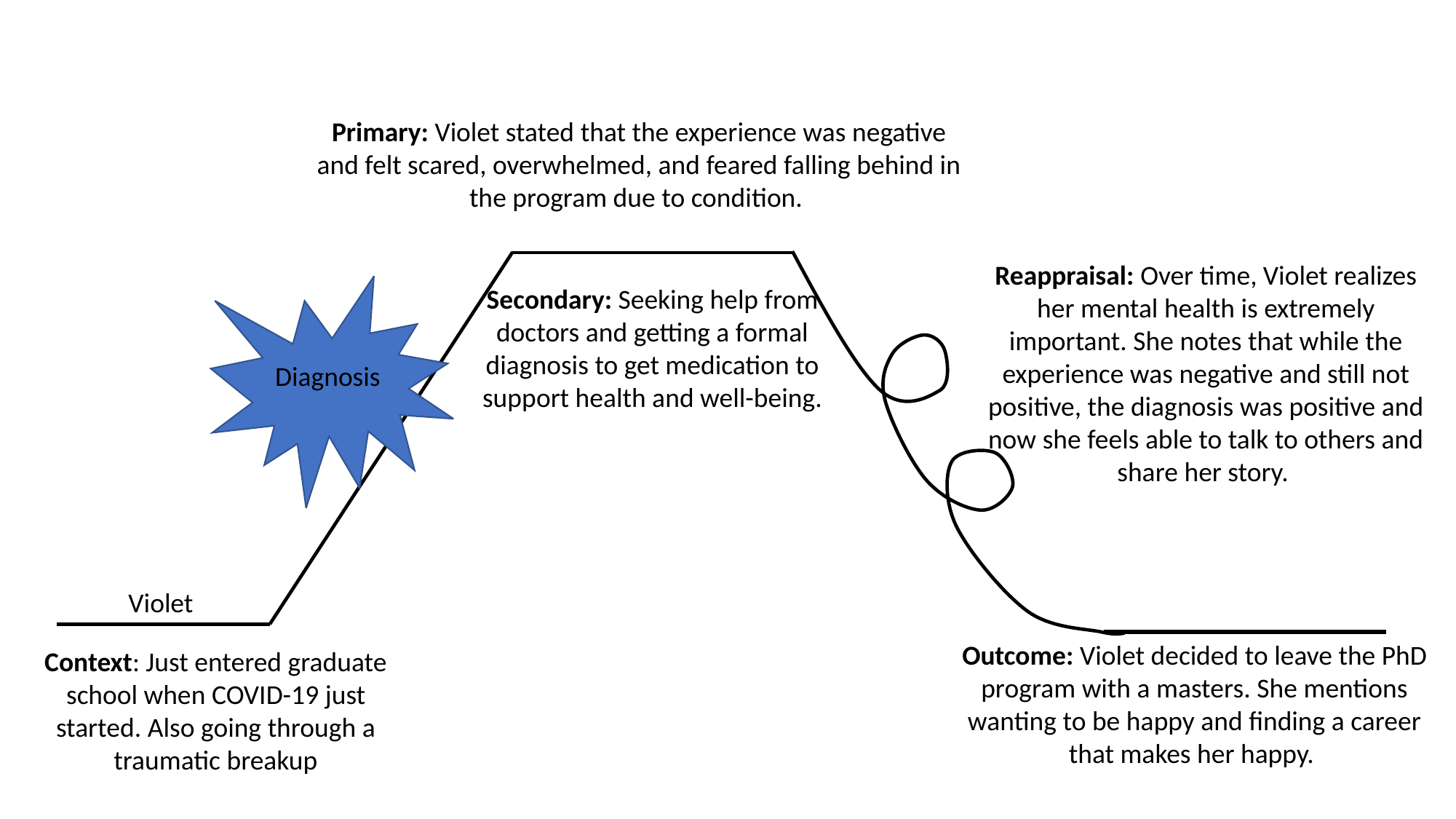

Primary: Violet stated that the experience was negative and felt scared, overwhelmed, and feared falling behind in the program due to condition.
Reappraisal: Over time, Violet realizes her mental health is extremely important. She notes that while the experience was negative and still not positive, the diagnosis was positive and now she feels able to talk to others and share her story.
Secondary: Seeking help from doctors and getting a formal diagnosis to get medication to support health and well-being.
Diagnosis
Violet
Outcome: Violet decided to leave the PhD program with a masters. She mentions wanting to be happy and finding a career that makes her happy.
Context: Just entered graduate school when COVID-19 just started. Also going through a traumatic breakup

## Slide 24
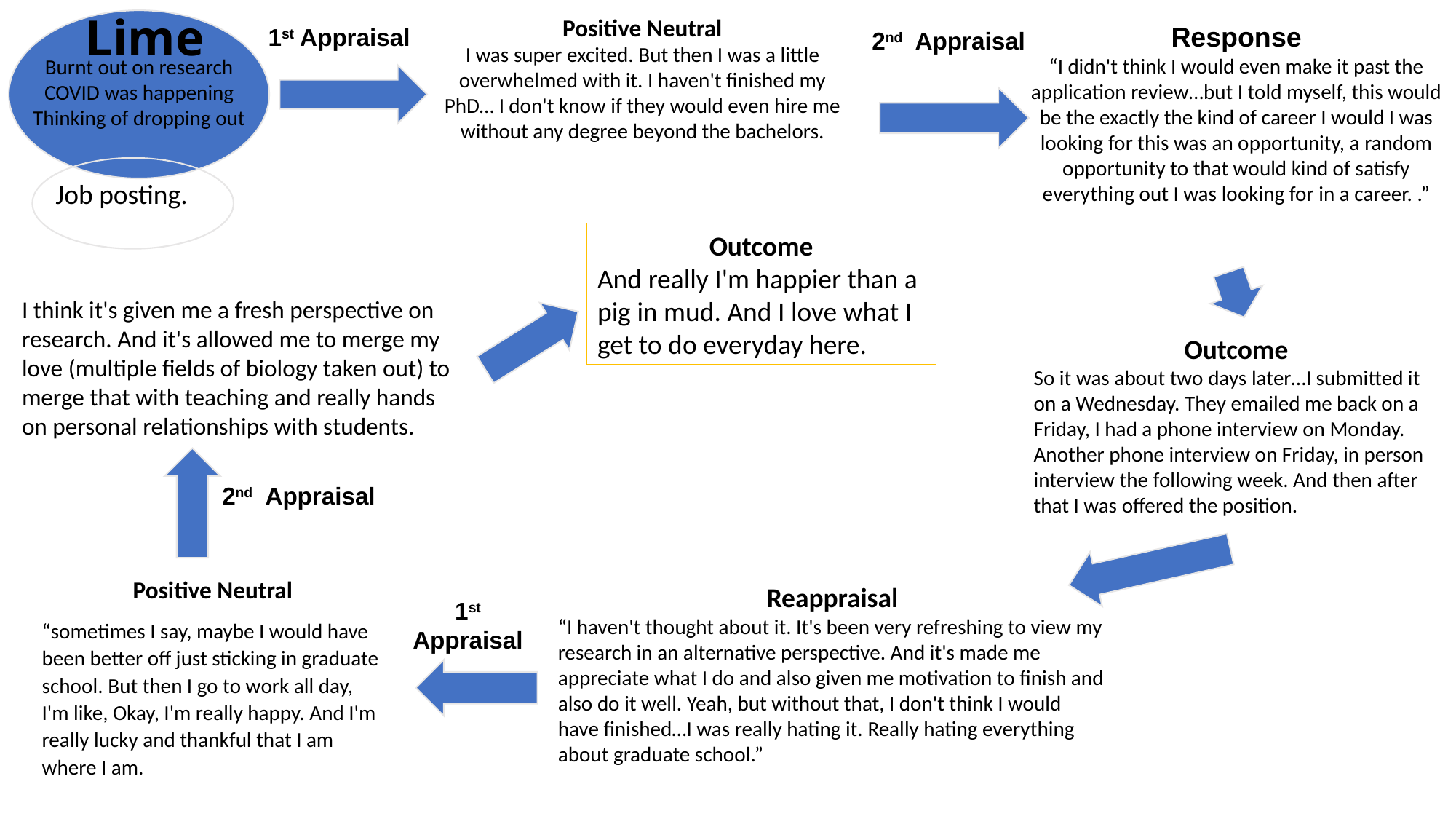

Lime
Positive Neutral
I was super excited. But then I was a little overwhelmed with it. I haven't finished my PhD… I don't know if they would even hire me without any degree beyond the bachelors.
Response
“I didn't think I would even make it past the application review…but I told myself, this would be the exactly the kind of career I would I was looking for this was an opportunity, a random opportunity to that would kind of satisfy everything out I was looking for in a career. .”
1st Appraisal
2nd Appraisal
Burnt out on research
COVID was happening
Thinking of dropping out
Job posting.
Outcome
And really I'm happier than a pig in mud. And I love what I get to do everyday here.
I think it's given me a fresh perspective on research. And it's allowed me to merge my love (multiple fields of biology taken out) to merge that with teaching and really hands on personal relationships with students.
Outcome
So it was about two days later…I submitted it on a Wednesday. They emailed me back on a Friday, I had a phone interview on Monday. Another phone interview on Friday, in person interview the following week. And then after that I was offered the position.
2nd Appraisal
Positive Neutral
“sometimes I say, maybe I would have been better off just sticking in graduate school. But then I go to work all day, I'm like, Okay, I'm really happy. And I'm really lucky and thankful that I am where I am.
Reappraisal
“I haven't thought about it. It's been very refreshing to view my research in an alternative perspective. And it's made me appreciate what I do and also given me motivation to finish and also do it well. Yeah, but without that, I don't think I would have finished…I was really hating it. Really hating everything about graduate school.”
1st Appraisal

## Slide 25
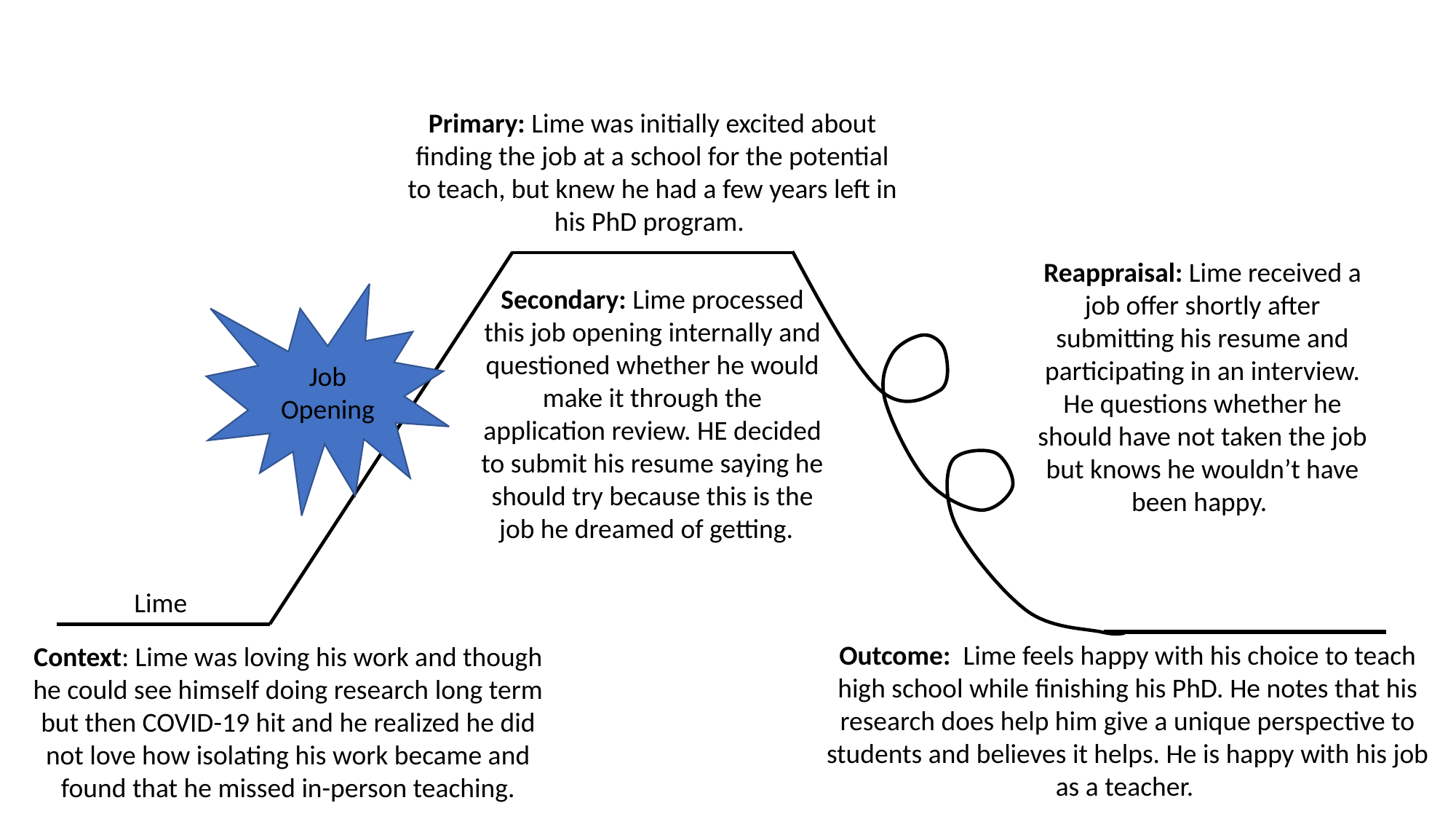

Primary: Lime was initially excited about finding the job at a school for the potential to teach, but knew he had a few years left in his PhD program.
Reappraisal: Lime received a job offer shortly after submitting his resume and participating in an interview. He questions whether he should have not taken the job but knows he wouldn’t have been happy.
Secondary: Lime processed this job opening internally and questioned whether he would make it through the application review. HE decided to submit his resume saying he should try because this is the job he dreamed of getting.
Job Opening
Lime
Outcome: Lime feels happy with his choice to teach high school while finishing his PhD. He notes that his research does help him give a unique perspective to students and believes it helps. He is happy with his job as a teacher.
Context: Lime was loving his work and though he could see himself doing research long term but then COVID-19 hit and he realized he did not love how isolating his work became and found that he missed in-person teaching.

## Slide 26
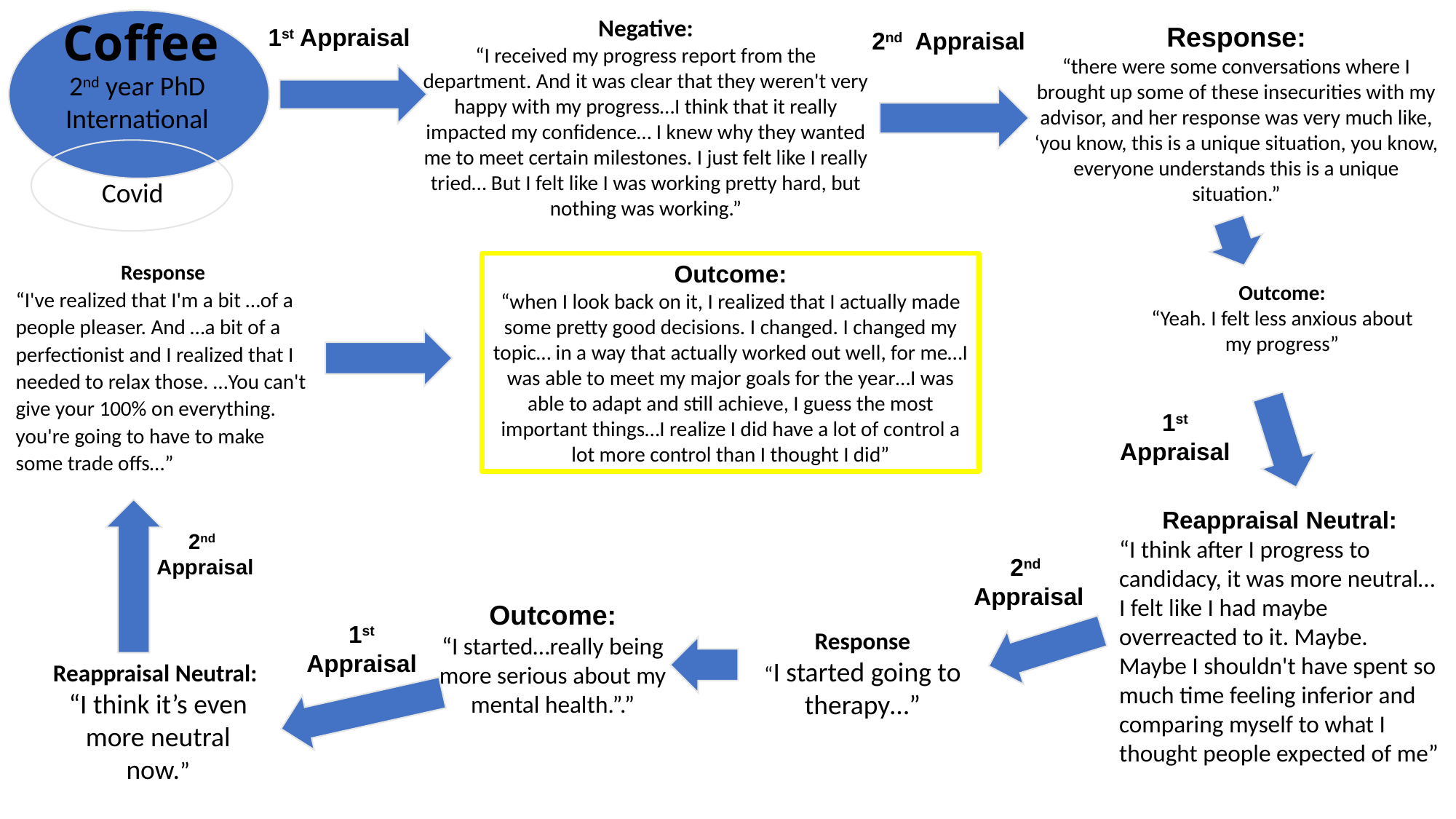

Coffee
Negative:
“I received my progress report from the department. And it was clear that they weren't very happy with my progress…I think that it really impacted my confidence… I knew why they wanted me to meet certain milestones. I just felt like I really tried… But I felt like I was working pretty hard, but nothing was working.”
Response:
“there were some conversations where I brought up some of these insecurities with my advisor, and her response was very much like, ‘you know, this is a unique situation, you know, everyone understands this is a unique situation.”
1st Appraisal
2nd Appraisal
2nd year PhD
International
Covid
Outcome:
“when I look back on it, I realized that I actually made some pretty good decisions. I changed. I changed my topic… in a way that actually worked out well, for me…I was able to meet my major goals for the year…I was able to adapt and still achieve, I guess the most important things…I realize I did have a lot of control a lot more control than I thought I did”
Response
“I've realized that I'm a bit …of a people pleaser. And …a bit of a perfectionist and I realized that I needed to relax those. …You can't give your 100% on everything. you're going to have to make some trade offs…”
Outcome:
“Yeah. I felt less anxious about my progress”
1st Appraisal
Reappraisal Neutral:
“I think after I progress to candidacy, it was more neutral…I felt like I had maybe overreacted to it. Maybe. Maybe I shouldn't have spent so much time feeling inferior and comparing myself to what I thought people expected of me”
2nd Appraisal
2nd Appraisal
Outcome:
“I started…really being more serious about my mental health.”.”
1st Appraisal
Response
“I started going to therapy…”
Reappraisal Neutral:
“I think it’s even more neutral now.”

## Slide 27
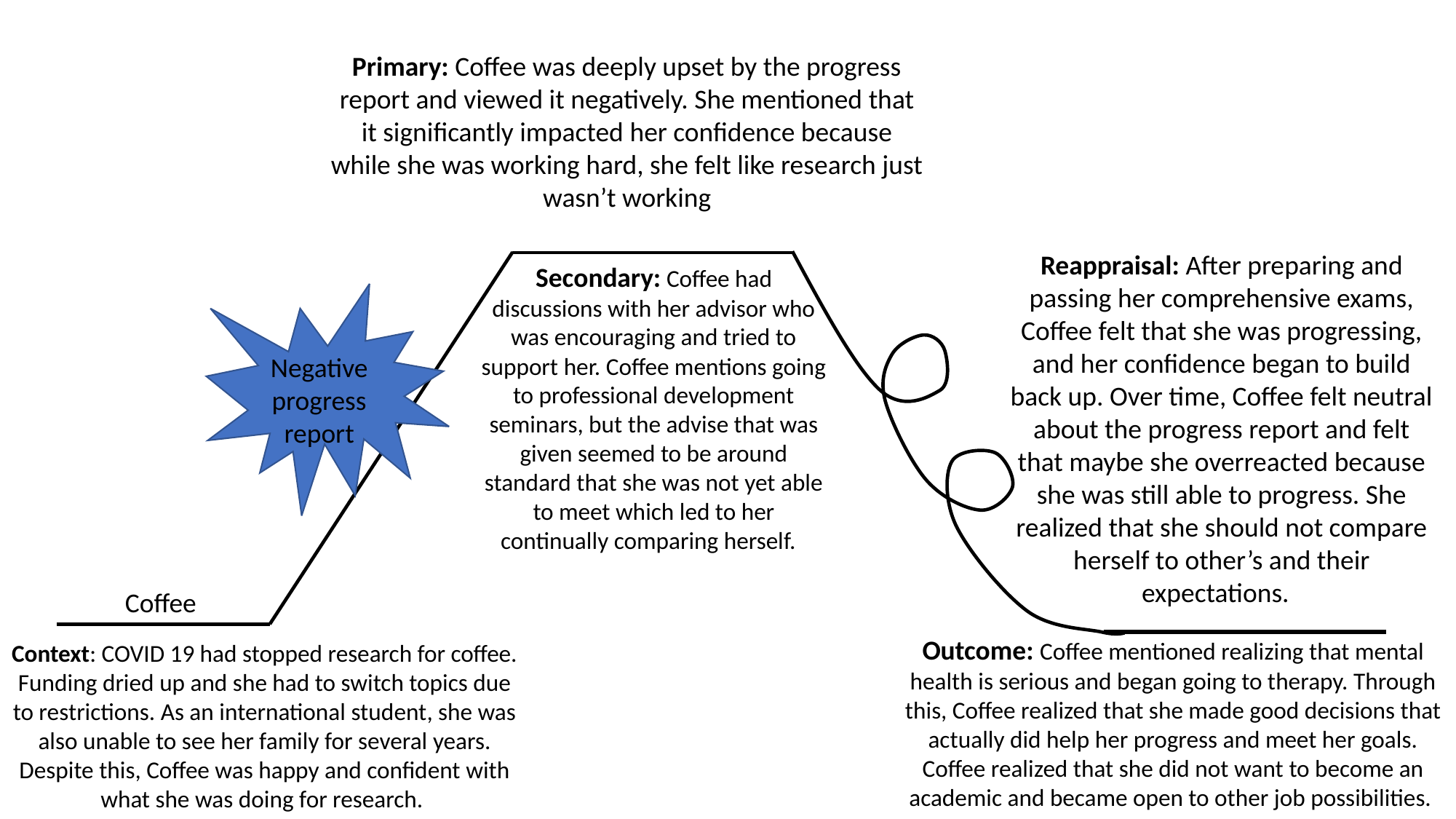

Primary: Coffee was deeply upset by the progress report and viewed it negatively. She mentioned that it significantly impacted her confidence because while she was working hard, she felt like research just wasn’t working
Reappraisal: After preparing and passing her comprehensive exams, Coffee felt that she was progressing, and her confidence began to build back up. Over time, Coffee felt neutral about the progress report and felt that maybe she overreacted because she was still able to progress. She realized that she should not compare herself to other’s and their expectations.
Secondary: Coffee had discussions with her advisor who was encouraging and tried to support her. Coffee mentions going to professional development seminars, but the advise that was given seemed to be around standard that she was not yet able to meet which led to her continually comparing herself.
Negative progress report
Coffee
Outcome: Coffee mentioned realizing that mental health is serious and began going to therapy. Through this, Coffee realized that she made good decisions that actually did help her progress and meet her goals. Coffee realized that she did not want to become an academic and became open to other job possibilities.
Context: COVID 19 had stopped research for coffee. Funding dried up and she had to switch topics due to restrictions. As an international student, she was also unable to see her family for several years. Despite this, Coffee was happy and confident with what she was doing for research.
