# Supplementary material for: A qualitative study of graduate student emotional and cognitive processing of unexpected (chance) events
Source: PLoS One. 2025 Aug 28;20(8):e0331182. doi: 10.1371/journal.pone.0331182 (PMC12393738; doi:10.1371/journal.pone.0331182)
Supplement: Attachment — Submitted filename: PONE-D-25-11301.docx [file pone.0331182.s005.docx]

Thank you for the opportunity to review manuscript PONE-D-25-11301 titled “Processing the Unexpected: A Qualitative Study of Graduate Student Appraisals of Chance Events.” The topic of stressors and needs for supports for students enrolled in post-secondary education is timely and of great interest to many. Overall, the manuscript is well written and organized, the appendices and supplementary files are informative and well done - however revisions are required.

- I was unfamiliar with the term “chance events” within the context of graduate studies. It likely explains why I was not sure what the study was about until I began reading the manuscript. Because of this, I wonder if the authors should reconsider the title of the paper so its relevance is apparent to those interested in supporting graduate students.
- A major issue I have with the manuscript pertains to its focus on students’ careers. At times the authors discuss students’ careers, while other times it is career intentions, career trajectories, and career outcomes. Given participants were enrolled in either master or PhD graduate programs, it appears the only thing that would be relevant is career intentions – and maybe academic goals. The authors are encouraged to carefully consider what the main concept of interest is. In keeping with this, line 389 seems to suggest participants may have been focusing on their career goals (which is different than academic goals). Further to this, on page 8 it states impact on attrition in graduate school.
- The manuscript is quite lengthy. While there are places where content is repeated and/or could be better synthesized, there are other areas where important information is missing. For example, on page 10 additional detail on how the authors selected participants from the potential pool of students, and ages of participants (if available) would also help readers determine the generalizability of findings. Ethical considerations are also dense and deserve some additional details. In places the discussion repeats information already reported in the findings.
- The limitations of the Appraisal Theory should be addressed, namely its reliance on individual cognitive appraisal, which is inherently subjective - should be included in the limitation section.
- Minor issue – there is some switching back and forth between current and past tense. Authors should do a careful review of the manuscript to ensure consistency in tenses.
- Page 8-9 identifies that half participants changed careers during graduate school while the other half changed careers after grad school. It is possible that career changes after graduate school were the result of other events or opportunities (and not something that took place in graduate school). This possibility should be addressed, especially since the researchers selected 10 participants from a larger group. Was this intentional to have students who had career changes after graduation? (this comment is similar to a previous one I provided).
- Additional detail is required on the recruitment and sampling technique. Specifically, description of the purposive sampling technique is needed. There are only 2 non-white participants and 2 Master’s students – was this intentional?
- Page 12 states “participants were provided pseudonyms based on color” – does this imply color of their skin? It is not clear what colors the authors are referring to. Or is it a case the pseudonyms are random colors selected to represent particpants?
- Was data from the draft interview protocol used for the 4 interviews included in the final study?
- Line 224-226 – it is not clear what is meant by “participants were guided that the event must be unexpected or unplanned” – does this mean the interviewer guided the participants i.e. tried to alter their perceptions of the event?
- Lines 268-269 – should this refer to career aspiration?
- Line 278 – suggestion writing “Research Question” out in full to be consistent with how this is presented further down in the manuscript.
- Line 530 the theme resiliency – I am not sure the illustrations provided supports resiliency.
- Line 659 The link between the study findings and career choice satisfaction among undergraduates needs to be established.
